# Persistence, chronicity, and recurrence of infection-associated urticaria following viral infections in children and adults: a systematic review

**DOI:** 10.3389/falgy.2026.1847423

**Published:** 2026-05-26

**Authors:** Saad Alhumaid, Sara Mohammad Alshehri, Zainah Sabr, Fatima A. Aborshaid, Sundus Noorsaeed, Ashwag A. Alsaidalani, Shorooq Shwgi Banjar, Karimah Aljasem, Fai A. AlQahtani, Sarah A. Baltyour, Ali Jawad Bu subaih, Ahmed Abdulmohsin Alwesaibi, Ali Hussain Alghanim, Zainab Al Alawi, Dalal Saadoun Alsaadoun

**Affiliations:** 1 School of Pharmacy, University of Tasmania, Hobart, TAS, Australia; 2 Department of Pediatrics, College of Medicine, Imam Mohammed Ibn Saud Islamic University, Riyadh, Saudi Arabia; 3 Imam Abdulrahman Al-Faisal Hospital, C1 Riyadh Health Cluster, Ministry of Health, Riyadh, Saudi Arabia; 4 Department of Pediatric Allergy, Asthma and Clinical Immunology, Dr. Sulaiman Al-Habib Hospital, Al Khobar, Saudi Arabia; 5 Department of Pediatrics, Faculty of Medicine, King Abdulaziz University, Jeddah, Saudi Arabia; 6 Department of Internal Medicine, Faculty of Medicine, King Abdulaziz University, Jeddah, Saudi Arabia; 7 Pediatrics Department, King Faisal General Hospital, Al-Ahsa Health Cluster, Ministry of Health, Al-Ahsa, Saudi Arabia; 8 Department of Pediatrics, College of Medicine, Imam Abdulrahman Bin Faisal University, Dammam, Saudi Arabia; 9 Infection Prevention and Control Department, Alomran General Hospital, Al-Ahsa Health Cluster, Ministry of Health, Al-Ahsa, Saudi Arabia; 10 College of Medicine, Dar Al Uloom University, Riyadh, Saudi Arabia; 11 Division of Allergy and Immunology, College of Medicine, King Faisal University, Al-Ahsa, Saudi Arabia; 12 Department of Internal Medicine, College of Medicine, King Faisal University, Al-Ahsa, Saudi Arabia

**Keywords:** adults, children, chronic spontaneous urticaria, infection-associated urticaria, long-term outcomes, persistence, recurrence, systematic review

## Abstract

**Background:**

Viral infections are recognized triggers of acute urticaria; however, the long-term outcomes of infection-associated urticaria, including persistence, recurrence, and progression to chronic disease, remain incompletely characterized.

**Objective:**

To systematically review the available evidence on the persistence, chronicity, and recurrence of infection-associated urticaria following viral infections in children and adults.

**Methods:**

A systematic review was conducted in accordance with PRISMA 2020 guidelines and prospectively registered in PROSPERO (CRD420261319656). Electronic databases (PubMed, Embase, CINAHL, Scopus, and Web of Science) were searched from inception to 17 March 2026. Observational studies reporting long-term outcomes of infection-associated urticaria following viral infection were included. Data extraction and risk of bias assessment using the Newcastle–Ottawa Scale were performed independently by two reviewers. Due to substantial heterogeneity, findings were synthesized narratively.

**Results:**

Five studies involving 596 participants were included. Most cases of infection-associated urticaria occurred during the acute phase of infection and resolved within weeks to months. However, persistence beyond 6 months was reported in up to 9.5% of cases, and a small proportion demonstrated persistent symptoms extending beyond 1 year. Reported recurrence and chronicity rates ranged from approximately 7% to 30%, although several estimates were derived from mixed-etiology cohorts in which infection-related cases were not analyzed separately. Delayed-onset urticaria, particularly following SARS-CoV-2 infection, was associated with an increased likelihood of progression to chronic spontaneous urticaria. However, interpretation is limited by the inclusion of mixed-etiology cohorts in which viral infection was not always laboratory-confirmed or analyzed separately.

**Conclusion:**

Infection-associated urticaria is typically self-limiting; however, a small but potentially clinically relevant subset of patients may develop persistent or chronic symptoms. Recognition of potential risk patterns, particularly delayed-onset urticaria in the context of SARS-CoV-2 infection, may support improved patient counselling and follow-up. Current evidence remains limited by heterogeneous study designs, mixed aetiologies, and inconsistent pathogen-specific reporting. Further prospective studies with standardized definitions and pathogen-specific analyses are needed.

**Systematic Review Registration:**

https://www.crd.york.ac.uk/prospero/display_record.php?RecordID=1319656, identifier CRD420261319656.

## Introduction

Urticaria is a common dermatological condition characterized by transient wheals, angioedema, or both, and is broadly classified as acute (< 6 weeks) or chronic (≥ 6 weeks) based on symptom duration (1, 2). Acute urticaria (AU) is frequently triggered by infections, medications, and food allergens, whereas chronic urticaria, particularly chronic spontaneous urticaria (CSU), is typically considered idiopathic or immune-mediated in origin (1, 2).

Viral infections are among the most commonly reported triggers of acute urticaria, particularly in pediatric populations. Respiratory and gastrointestinal infections have been consistently implicated, and urticaria often occurs concurrently with or shortly after infection onset (1, 2). In most cases, infection-associated urticaria follows a self-limiting course, resolving within days to weeks (3, 4). However, emerging evidence suggests that a subset of patients may experience prolonged disease, including persistence beyond the acute phase, recurrence after initial resolution, or progression to chronic urticaria (4–8).

The long-term trajectory of infection-associated urticaria remains poorly defined. While acute infection-associated urticaria is well recognized, fewer studies have systematically evaluated outcomes such as persistence, chronicity, and recurrence beyond the initial episode (4–8). Existing literature is heterogeneous, with variation in study design, patient populations, definitions of outcomes, and duration of follow-up (4–8). Furthermore, many studies include mixed-etiology cohorts in which viral infection is not the exclusive trigger, limiting the ability to attribute long-term outcomes specifically to viral causes (5, 7).

Importantly, much of the available literature on infection-associated urticaria derives from mixed-etiology acute urticaria cohorts in which infections represent one of several potential triggers (5, 7). In many studies, viral pathogens are not laboratory-confirmed and long-term outcomes are not stratified according to infectious aetiology, limiting direct attribution of persistence or chronicity specifically to post-viral urticaria. These limitations complicate interpretation of the available evidence and highlight the need for cautious synthesis of current findings (4, 5, 7).

Recent interest in post-infectious sequelae, particularly following SARS-CoV-2 infection, has further highlighted the potential for delayed or prolonged dermatological manifestations, including urticaria. In this context, delayed-onset of urticaria and progression to CSU have been reported, raising questions about underlying immunological mechanisms and long-term disease risk (6). However, the available evidence remains limited and fragmented (4–8).

Previous studies have primarily focused on the acute phase of urticaria and short-term outcomes. For example, Lin et al. reported that infections accounted for 45.2% of acute urticaria cases in children and were associated with longer symptom duration, although follow-up was limited to the acute phase and did not capture long-term outcomes (3). Consequently, there is a need for a comprehensive synthesis of available evidence addressing the longer-term clinical course of infection-associated urticaria following viral infections.

Previous reviews addressing urticaria and viral infections have primarily focused on acute clinical manifestations, diagnostic considerations, and associations with specific viral pathogens, particularly SARS-CoV-2 infection (9, 10). However, these reviews generally did not specifically evaluate long-term outcomes such as persistence, recurrence, or progression to chronic disease across pediatric and adult populations. Accordingly, the present review differs by specifically focusing on the longer-term clinical trajectory of infection-associated urticaria and the available evidence relating to chronicity and recurrence following viral infections.

To our knowledge, no prior systematic review has specifically synthesized long-term outcomes of infection-associated urticaria following viral infections across both pediatric and adult populations, including persistence, recurrence, and progression to chronic disease.

Therefore, this systematic review aims to evaluate the persistence, chronicity, and recurrence of infection-associated urticaria following viral infections in children and adults, with a focus on characterizing long-term outcomes and identifying patterns that may inform clinical management and future research.

## Methods

### Study design and registration

This systematic review was conducted in accordance with the Preferred Reporting Items for Systematic Reviews and Meta-Analyses (PRISMA) 2020 guidelines (11). The PRISMA 2020 checklist is provided in the Supplementary Materials (11). The study protocol was prospectively registered in the International Prospective Register of Systematic Reviews (PROSPERO) under registration number CRD420261319656. Any deviations from the registered protocol were documented and justified where applicable.

### Search strategy

A comprehensive literature search was conducted to identify studies evaluating long-term outcomes of infection-associated urticaria in the context of viral infections. The following electronic databases were searched from database inception to 17 March 2026: PubMed, Embase, CINAHL, Scopus, and Web of Science.

Search strategies combined controlled vocabulary terms (e.g., Medical Subject Headings [MeSH] and database-specific indexing terms) with free-text keywords related to urticaria and viral infections. The search strategy incorporated MeSH terms and free-text keywords related to urticaria and viral infections, including specific terms for SARS-CoV-2 and COVID-19. Boolean operators (AND, OR) were used to combine search terms. Core search terms included urticaria, chronic urticaria, chronic spontaneous urticaria, hives, viral infection, virus, post-viral, persistence, recurrence, and long-term outcomes. The search strategy was adapted for each database to account for differences in indexing and search syntax. No restrictions were applied during the search; language eligibility was applied during the screening stage. The complete search strategy for all databases is provided in Supplementary Table S1.

Grey literature sources were not included in the search.

To ensure comprehensive coverage of the literature, the reference lists of included studies and relevant systematic reviews were manually screened. Backward and forward citation tracking was also performed to identify additional potentially eligible studies.

All retrieved records were imported into EndNote (version 21.2) for removal of duplicate citations and subsequently uploaded to Covidence for study screening and selection.

### Eligibility criteria

Studies were eligible for inclusion if they involved children or adults diagnosed with urticaria associated with or occurring after a viral infection and reported outcomes related to persistence, chronicity, or recurrence of urticaria. Studies in which viral infection was identified as a potential or predominant trigger, including mixed-etiology or multifactorial acute urticaria cohorts, were considered eligible provided that infection-associated urticaria was explicitly reported or clearly described within the study population or results. Because of the limited availability of pathogen-specific long-term outcome studies, mixed-etiology cohorts were included when infection-associated urticaria represented a clinically relevant component of the study population. These studies were interpreted cautiously during synthesis due to the inability to isolate outcomes exclusively attributable to viral infection. The review question and eligibility criteria were defined according to the PICOS framework (Population, Exposure, Comparator, Outcomes, and Study design).

Eligible study designs included observational studies such as cohort studies, case-control studies, cross-sectional studies, and case series. Case reports and small case series (< 5 participants) were excluded, while larger case series were included only if they reported relevant long-term outcomes. Only peer-reviewed articles published in English were considered.

Articles were excluded if they were review articles, editorials, commentaries, conference abstracts without full text, preprints, or other non-peer-reviewed reports. Studies that did not report outcomes related to long-term urticaria persistence, chronicity, or recurrence following viral infection were also excluded.

COVID-19-related case reports and small case series describing new-onset chronic spontaneous urticaria following SARS-CoV-2 infection were identified during screening; however, these studies were excluded because they did not meet the predefined eligibility criteria requiring larger observational studies and/or systematic reporting of long-term outcomes.

### Operational definitions

Long-term outcomes were defined as those extending beyond the acute phase of infection, with particular emphasis on chronic urticaria (≥ 6 weeks), persistence, or recurrence. Studies reporting only acute urticaria without follow-up beyond the initial illness were excluded, as they do not inform long-term disease trajectories.

Key outcomes were defined in accordance with established clinical definitions and international urticaria guidelines. Persistence was defined as the continuation of urticarial symptoms beyond the acute phase of the suspected or reported infectious trigger. Acute urticaria was defined as the presence of wheals, angioedema, or both lasting for < 6 weeks, whereas chronic urticaria was defined as symptoms persisting for ≥ 6 weeks (1, 2).

Chronic urticaria was further classified as chronic spontaneous urticaria (CSU), defined as the occurrence of wheals, angioedema, or both in the absence of a specific identifiable external trigger, consistent with international guideline definitions (1, 2).

Recurrence was defined as the reappearance of urticaria following a symptom-free interval, in line with terminology used in urticaria research and guideline-based frameworks (1, 2).

When studies used alternative definitions or did not explicitly distinguish between subtypes, the original study definitions were retained and interpreted within these standardized frameworks. Where necessary, outcomes were mapped to the predefined categories to ensure consistency across studies.

In studies with mixed aetiologies, outcomes were interpreted in the context of reported or predominant triggers, with particular consideration of cases in which viral infection was identified as a reported or primary contributing factor within the study.

### Study selection

All records retrieved from the database searches were imported into reference management software and duplicate records were removed. Two reviewers independently screened titles and abstracts for eligibility. Title and abstract screening and full-text review were performed within Covidence (Veritas Health Innovation, Melbourne, Australia). Full-text articles were subsequently assessed against predefined inclusion criteria. Disagreements were resolved through discussion and, when necessary, consultation with a third reviewer.

**Figure 1:**
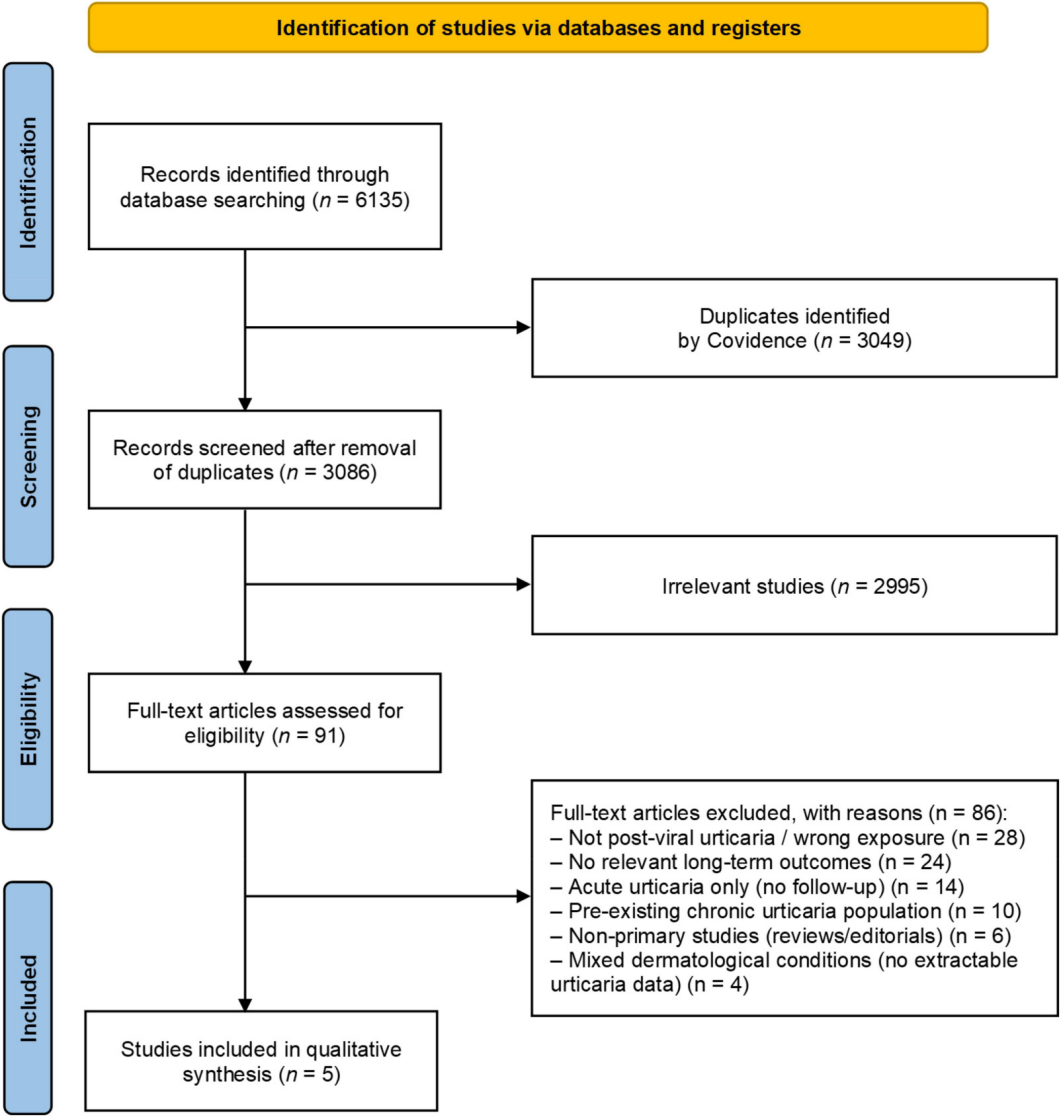
Flow diagram of studies included in the systematic review.

The study selection process is summarized using a PRISMA flow diagram (Figure 1). Reasons for exclusion at the full-text stage were recorded and are presented in Supplementary Table S2.

### Data extraction

Data extraction was performed independently by two reviewers using a standardized data extraction form. Extracted variables included first author, year of publication, country, study design, sample size, study population characteristics, viral pathogen, diagnostic methods for infection, type of urticaria, duration of symptoms, follow-up period, and outcomes related to persistence, chronicity, or recurrence. Any discrepancies between reviewers were resolved through discussion and consensus. If consensus could not be reached, a third reviewer was consulted.

When required data were unclear or incomplete, information was interpreted based on available study descriptions and reported data. Extracted data were cross-checked between reviewers prior to analysis.

### Risk of bias assessment

The methodological quality of included observational studies was evaluated using the Newcastle–Ottawa Scale (NOS) (12). This tool assesses study quality across three domains: selection of study participants, comparability of study groups, and outcome assessment. Studies scoring 7–9 points were considered low risk of bias, 4–6 moderate risk, and 0–3 high risk of bias (12). The detailed NOS scoring for each included study is provided in Supplementary Table S3.

### Data synthesis

Heterogeneity was assessed qualitatively based on study design, outcome definitions, viral pathogens, and follow-up duration. Given substantial clinical and methodological heterogeneity among included studies, including differences in viral pathogens, study populations, outcome definitions, and follow-up durations, a quantitative meta-analysis was not performed, as pooling of results would likely produce misleading estimates. Given the inclusion of mixed-etiology cohorts and inconsistent pathogen confirmation across studies, findings were interpreted as evidence relating primarily to infection-associated urticaria rather than definitively confirmed post-viral urticaria. Therefore, findings were synthesized using a narrative descriptive approach. Study characteristics and key outcomes were summarized in tables, and patterns related to persistence, chronicity, and recurrence of infection-associated urticaria were qualitatively described. Where studies included mixed aetiologies, findings were interpreted with caution, and the potential contribution of viral triggers was considered in the qualitative synthesis. Where sufficient information was available, findings were also described according to age group (children vs. adults) and viral pathogen.

## Results

### Study characteristics

A total of five studies met the inclusion criteria, comprising three prospective cohort studies, one retrospective case–control study, and one prospective observational study (4–8). Studies were conducted across diverse settings, including Europe, Asia, and the Middle East, and included both pediatric and adult populations (Table 1) (4–8).

**Table 1:** Characteristics of included studies evaluating infection-associated urticaria following viral infections.

First author (year)	Country	Study design	Sample size	Population (age group)	Viral pathogen	Diagnostic method	Type of urticaria	Follow-up duration
Aoki (1994) (4)	Japan	Prospective observational cohort	50 patients	Children and adults (3 months–88 years)	Infection-associated (predominantly presumed viral or bacterial infections; respiratory, gastrointestinal, and systemic symptoms reported)	Clinical assessment based on history of infection-related symptoms; no laboratory confirmation of specific viral pathogens	AU (infection-associated)	Up to 1 year
Cetinkaya (2019) (7)	Turkey	Prospective cohort study	83 children	Preschool-aged children (< 5 years)	Mixed infectious triggers, including viral infections (e.g., herpes simplex virus type 1, Epstein–Barr virus, Mycoplasma pneumoniae)	Clinical assessment supported by laboratory investigations (serology, microbiological testing)	AU (mixed aetiology, infection-associated subgroup)	Up to 2 years
Kara (2024) (6)	Turkey	Retrospective case–control study	92 patients	Adults (mean age ∼27–36 years; predominantly adults)	SARS-CoV-2 (COVID-19 infection)	PCR-confirmed COVID-19 infection (majority); clinical diagnosis in a minority	AU and CSU (post-COVID-19 infection)	Up to ∼22 months (CSU); acute cases ≤3 weeks
Mortureux (1998) (8)	France	Prospective cohort study	57 infants (40 with follow-up data)	Infants and young children (1–36 months)	Predominantly viral infections (e.g., adenovirus, Epstein–Barr virus, enterovirus, respiratory syncytial virus, rotavirus, varicella-zoster virus)	Clinical assessment supported by laboratory investigations (viral cultures, serology, hematological parameters)	AU (infection-associated)	1–2 years
Talarico (2021) (5)	Italy	Prospective observational cohort study	314 children	Children (< 18 years; predominantly preschool-aged)	Mixed infectious triggers^a^ (predominantly presumed viral infections; upper respiratory tract infections most common)	Clinical assessment based on history and physical examination; laboratory investigations performed selectively to identify underlying cause	AU (mixed aetiology, infection-associated subgroup)	1, 3, and 6 months

AU, acute urticaria; COVID-19, coronavirus disease 2019; CSU, chronic spontaneous urticaria; PCR, polymerase chain reaction; SARS-CoV-2, severe acute respiratory syndrome coronavirus 2.

a Infection identified in 43.9% of cases; outcomes not stratified by aetiology.

Sample sizes ranged from 50 to 314 participants (4–8). Most studies focused on pediatric populations, particularly infants and preschool-aged children (5, 7, 8), while one study evaluated predominantly adult patients following SARS-CoV-2 infection (6). Infectious triggers, including suspected or confirmed viral infections, were identified as either predominant or contributing factors across included studies; however, several cohorts included mixed aetiologies in which infections were not the exclusive cause (5, 7).

### Clinical characteristics and viral triggers

Across studies, urticaria most commonly occurred during the acute phase of infection, typically within days before or after the onset of infection-related symptoms (4, 7, 8). Reported infectious triggers included respiratory and gastrointestinal infections, herpes simplex virus type 1, Epstein–Barr virus, and SARS-CoV-2; however, pathogen confirmation was inconsistent across studies, and several cohorts relied primarily on clinical assessment rather than laboratory-confirmed viral diagnoses (Table 2) (4, 6–8).

**Table 2:** Reported infectious triggers and clinical characteristics associated with urticaria.

Study	Reported infectious trigger	Timing of urticaria (acute/post-viral)	Severity reported	Hospitalization	Key clinical features
Aoki (1994) (4)	Mixed infectious triggers, predominantly presumed viral infections (e.g., respiratory and gastrointestinal infections; no laboratory confirmation)	Acute (around onset of infection; symptoms typically occurred within days before or after infection onset)	Not formally classified; majority mild and self-limiting, with most cases resolving within 2 weeks	Not reported (outpatient cohort)	AU with pruritic wheals; majority associated with symptoms suggestive of infection (respiratory, gastrointestinal, or systemic); short disease duration in most cases; minority progressed to prolonged or persistent disease
Cetinkaya (2019) (7)	Mixed infectious triggers including viral infections (notably herpes simplex virus type 1; also Epstein–Barr virus and Mycoplasma pneumoniae)	Acute (during or concurrent with infection)	Mild to moderate in most cases; some cases with angioedema and rare anaphylaxis	Not consistently reported (patients recruited from pediatric allergy and emergency departments)	AU with pruritic wheals; angioedema (∼28%); occasional anaphylaxis (∼3.6%); upper respiratory tract infection common trigger; higher severity associated with longer symptom duration
Kara (2024) (6)	SARS-CoV-2 (COVID-19 infection)	Both acute (concurrent with infection) and post-viral (CSU onset typically > 2 weeks after infection)	Not formally classified; majority non-severe COVID-19; urticaria severity variable (CSU prolonged, AU self-limiting)	Not specifically reported; majority non-severe cases (implies predominantly outpatient)	AU occurred concurrently with infection and was self-limiting (∼1 week); CSU developed in a minority with delayed-onset (∼2–3 weeks post-infection) and prolonged duration (months); delayed-onset may predict chronicity; eosinopenia associated with CSU
Mortureux (1998) (8)	Predominantly viral infections (adenovirus, Epstein–Barr virus, enterovirus, respiratory syncytial virus, rotavirus, varicella-zoster virus)	Acute (during or concurrent with viral infection)	Moderate to severe (hospitalized cohort; frequent angioedema and systemic symptoms)	Yes (all patients hospitalized)	Generalized urticaria with annular/geographic lesions; angioedema (≈60%); fever (∼50%); respiratory symptoms (≈60%); arthralgia (∼30%); haemorrhagic lesions more frequent in infection-associated cases
Talarico (2021) (5)	Mixed infectious triggers^a^, predominantly presumed viral infections (upper respiratory tract infections most common; no systematic pathogen-specific confirmation)	Acute (during or concurrent with infection; onset assessed at emergency department presentation)	Mild (40.4%), moderate (44.5%), severe (14.9%)	Not reported (emergency department cohort; predominantly outpatient management)	AU with pruritic wheals; angioedema may occur; infections identified as the most common trigger (43.9%); majority of cases self-limiting; a minority progressed to persistence or recurrence at follow-up

AU, acute urticaria; COVID-19, coronavirus disease 2019; CSU, chronic spontaneous urticaria; SARS-CoV-2, severe acute respiratory syndrome coronavirus 2.

a Infection identified in 43.9% of cases; outcomes not stratified by aetiology.

Clinical presentation was generally consistent with acute urticaria, characterized by pruritic wheals with or without angioedema (4, 5, 7, 8). Most cases were mild to moderate in severity and self-limiting (4, 5, 7). In hospitalized cohorts, more severe presentations were observed, including systemic symptoms such as fever and arthralgia (8). Angioedema was reported in a substantial proportion of pediatric cases, particularly in infection-associated cohorts (7, 8).

### Persistence and progression to chronic disease

Outcomes related to persistence, chronicity, and recurrence are summarized in Table 3. Most reported cases of infection-associated urticaria resolved within weeks to months (4, 5). In the study by Aoki et al., 86% of cases resolved within 2 weeks and 96% within 3 months; however, a small proportion (approximately 4%) demonstrated persistence beyond 1 year, suggesting progression to chronic disease (4).

**Table 3:** Reported outcomes of persistence, chronicity, and recurrence in infection-associated urticaria.

Study	Outcome type	Definition used	Outcome measure	Outcome attribution	Follow-up	Key findings
Aoki (1994) (4)	Persistence and chronicity	Persistence defined as continued urticaria beyond the acute phase; chronicity inferred when symptoms persisted beyond typical resolution periods (≥ 6 weeks not explicitly defined by authors)	Duration of urticaria and proportion of patients with prolonged symptoms	Predominantly presumed infection-associated cohort; no pathogen-specific stratification	Up to 1 year	Majority of cases resolved within 2 weeks (86%) and within 3 months (96%); a small proportion (2/50, ∼4%) demonstrated prolonged persistence beyond 1 year, suggesting progression to chronic or persistent urticaria.
Cetinkaya (2019) (7)	Chronicity and recurrence	Chronic urticaria defined as symptoms persisting ≥ 6 weeks; recurrence defined as reappearance of urticaria after a symptom-free interval (as per study definitions)	Proportion of patients developing recurrent or chronic urticaria	Whole mixed-etiology cohort; infection-associated cases not analyzed separately	Up to 2 years	Recurrence occurred in 28 of 83 patients (33.7%), and chronic urticaria developed in 6 of 83 patients (7.2%).
Kara (2024) (6)	Persistence and chronicity	CSU defined as urticaria persisting for ≥ 6 weeks following COVID-19 infection; AU defined as symptoms resolving within < 6 weeks	Duration of urticaria and proportion of patients developing CSU following COVID-19 infection	SARS-CoV-2-specific cohort	Up to 22 months (CSU cases); acute urticaria duration up to ∼3 weeks	CSU occurred in a minority (7/92) with prolonged duration (approximately 7.5 months), whereas AU resolved within approximately 1 week; delayed-onset (> 2 weeks post-infection) was associated with progression to chronicity.
Mortureux (1998) (8)	Chronicity and recurrence	Chronic urticaria defined as symptoms persisting ≥ 6 weeks; recurrence defined as reappearance of urticaria after initial resolution (as per study definitions aligned with clinical practice)	Proportion of patients developing chronic or recurrent urticaria	Predominantly presumed viral infection-associated cohort	1–2 years	Approximately 20–30% (based on 40 patients with available follow-up data) developed chronic or recurrent urticaria at follow-up.
Talarico (2021) (5)	Persistence and recurrence	Persistence defined as continued presence of urticaria at follow-up visits (1, 3, and 6 months); recurrence defined as reappearance of urticaria after initial resolution (as per study follow-up assessments)	Proportion of patients with persistence and recurrence of urticaria at follow-up	Whole mixed-etiology cohort; outcomes not stratified according to infectious trigger	1, 3, and 6 months	Based on available follow-up data, recurrence occurred in approximately 34/314 patients (10.8%) at 1 month, 35/314 patients (11%) at 3 months, and 30/314 patients (9.5%) at 6 months; persistence of urticaria was observed in approximately 30/314 patients (9.5%) at 6 months. Outcomes were reported for a mixed-etiology cohort and were not stratified according to viral trigger.

AU, acute urticaria; COVID-19, coronavirus disease 2019; CSU, chronic spontaneous urticaria.

Outcome attribution refers to whether reported long-term outcomes were derived from pathogen-specific cohorts, infection-associated subgroups, or whole mixed-etiology study populations.

Percentages and numerators/denominators are reported as provided in the original studies where available.

Where applicable, outcome estimates were based on available follow-up data reported in the original studies.

Similarly, Talarico et al. reported persistence of urticaria in 9.5% of patients at 6 months, although outcomes were derived from a mixed-etiology cohort and were not stratified by viral trigger (5). These findings indicate that while the majority of cases are self-limiting, a subset of patients may experience prolonged disease beyond the acute phase.

### Recurrence and chronicity

Recurrent and chronic urticaria were reported across several studies, although estimates varied substantially. In the prospective cohort by Mortureux et al., approximately 20%–30% of patients developed recurrent or chronic urticaria during 1–2 years of follow-up (8). Similarly, Cetinkaya et al. reported recurrence in 33.7% and chronic urticaria in 7.2% of children; however, these findings were derived from a mixed-etiology cohort in which infections accounted for approximately half of cases and outcomes were not stratified according to infectious trigger (7).

In the context of SARS-CoV-2 infection, Kara et al. reported that chronic spontaneous urticaria developed in a minority of patients (7/92), with a mean duration of approximately 7.5 months (6). Notably, delayed-onset of urticaria following infection (defined as > 2 weeks in the included SARS-CoV-2 study) was associated with progression to chronic disease (6).

### Timing and predictors of chronicity

The temporal relationship between infection and urticaria onset may influence disease trajectory; however, available evidence remains limited and is derived primarily from a small number of heterogeneous studies (4, 6). Urticaria occurring concurrently with infection was generally self-limiting (4), whereas delayed-onset urticaria, particularly following SARS-CoV-2 infection, was associated with an increased likelihood of chronicity (6).

Although formal predictive modelling was limited, several factors were associated with prolonged or recurrent disease, including greater initial severity, presence of angioedema, and infection-related triggers (4, 7). However, these findings should be interpreted with caution given the heterogeneity of study designs and lack of consistent adjustment for confounding factors.

### Summary of findings

Overall, infection-associated urticaria was generally reported as an acute and self-limiting condition; however, a small but potentially clinically relevant subset of patients appeared to develop persistent, recurrent, or chronic disease (4–8). Reported rates of progression varied substantially across studies, reflecting differences in study populations, outcome definitions, follow-up duration, and methods of identifying infectious triggers (4–8). Importantly, several included studies evaluated mixed-etiology cohorts without pathogen-specific outcome stratification, limiting direct attribution of long-term outcomes specifically to viral infection (5, 7).

### Risk of bias

The risk of bias assessment for included studies is presented in Table 4. Overall, all included studies were assessed as having moderate risk of bias based on the Newcastle–Ottawa Scale.

**Table 4:** Risk of bias assessment using the Newcastle–Ottawa scale (NOS).

Study	Selection (0–4)	Comparability (0–2)	Outcome (0–3)	Total score (0–9)	Risk of bias
Aoki (1994) (4)	3	0	2	5	Moderate
Cetinkaya (2019) (7)	3	1	2	6	Moderate
Kara (2024) (6)	3	1	2	6	Moderate
Mortureux (1998) (8)	3	0	2	5	Moderate
Talarico (2021) (5)	3	1	2	6	Moderate

## Discussion

This systematic review synthesizes the available evidence on long-term outcomes of infection-associated urticaria following viral infections in children and adults, while acknowledging important limitations related to mixed-etiology cohorts and inconsistent pathogen-specific reporting. Overall, the available evidence indicates that infection-associated urticaria is generally an acute, self-limiting condition; however, a small but potentially clinically relevant subset of patients may experience prolonged or recurrent disease, with progression to chronic urticaria in a minority of cases (4–8). Collectively, these observations provide preliminary and hypothesis-generating insights into the long-term trajectory of infection-associated urticaria; however, given the limited and heterogeneous evidence base, they should be interpreted with caution. The findings may nevertheless have implications for patient counselling, follow-up, and risk stratification.

Across included studies, most cases resolved within weeks to months, consistent with the typical natural history of acute urticaria (4, 5). Nevertheless, persistence beyond the acute phase was observed in a subset of patients, including reports of symptoms extending beyond 6 months and, in some cases, beyond 1 year (4, 5). From a clinical perspective, recognizing this minority of patients at risk of chronicity is important, as early identification may influence follow-up and management decisions.

Recurrence and chronicity were variably reported, with estimates ranging from approximately 7% to 30% depending on study design, population, and follow-up duration (5, 6, 8). Notably, higher rates of recurrence and chronicity were often observed in pediatric cohorts, reflecting the predominance of infection-associated triggers in early childhood (5, 7, 8). However, interpretation of these findings is limited by the inclusion of mixed-etiology populations in several studies, in which viral infection was not the exclusive trigger and outcomes were not consistently stratified by aetiology (5, 7).

The temporal relationship between infection and urticaria onset may be clinically relevant. Urticaria occurring concurrently with infection was generally self-limiting (4), whereas delayed-onset, particularly following SARS-CoV-2 infection, was associated with an increased likelihood of chronicity (6). However, the > 2-week threshold for defining delayed-onset is derived from a single study and is not based on standardized criteria; therefore, this observation should be interpreted cautiously and considered hypothesis-generating. This may reflect differences in underlying immunological mechanisms between acute infection-associated urticaria and post-infectious or immune-mediated disease, although further mechanistic studies are needed.

Importantly, findings related to delayed-onset urticaria and progression to chronic disease are largely derived from studies of SARS-CoV-2 infection and should not be generalized to viral infections more broadly. Accordingly, evidence derived from pathogen-specific cohorts, particularly SARS-CoV-2 studies, should be interpreted separately from indirect evidence obtained from mixed-etiology acute urticaria populations without pathogen-stratified outcome reporting. SARS-CoV-2 is associated with unique immunological features, including post-infectious immune dysregulation and autoimmune phenomena, which may contribute to differences in disease trajectory compared with other viral pathogens (13, 14). In this context, it is important to distinguish between acute infection-associated urticaria occurring concurrently with viral illness, post-infectious or delayed-onset urticaria (particularly described in COVID-19), and chronic spontaneous urticaria potentially triggered by infection. These entities likely represent distinct clinical and pathophysiological processes, consistent with current urticaria classification frameworks (1, 2), and the available evidence does not support treating them as a single homogeneous condition.

A similar temporal pattern of delayed-onset urticaria predicting chronicity has not been clearly established in autoimmune or autoinflammatory diseases. Instead, prior literature suggests that these conditions are relevant to chronic urticaria in different ways. Chronic spontaneous urticaria is increasingly recognized as an immune-mediated disorder with autoimmune endotypes and autoimmune comorbidities (15), whereas autoinflammatory syndromes such as cryopyrin-associated periodic syndromes and Schnitzler syndrome may present with chronic urticaria-like eruptions and systemic inflammation (16). These disorders are therefore better considered distinct clinical and pathophysiological entities rather than direct analogues of post-COVID-19 delayed-onset urticaria.

An important limitation across the available literature is the difficulty in attributing long-term urticaria outcomes specifically to viral infection (4, 5, 7). Several included studies evaluated mixed-etiology acute urticaria cohorts in which infections represented one of multiple potential triggers, including medications, foods, or idiopathic causes (5, 7). Furthermore, many studies relied primarily on clinical assessment without systematic laboratory confirmation of viral pathogens (4, 5). In most studies, long-term outcomes were not stratified according to infectious aetiology, limiting the ability to determine whether reported persistence, recurrence, or chronicity rates were directly attributable to viral infection itself (5, 7).

Several studies highlighted the complexity of attributing urticaria specifically to viral infection. In real-world clinical settings, infection-related urticaria frequently coexists with other potential triggers, including medications such as antibiotics (5). As a result, distinguishing between infection-induced and drug-induced urticaria may be challenging, and residual confounding cannot be excluded (4, 5).

The predominance of pediatric populations in the included studies underscores important age-related differences in disease presentation and triggers. Infection-associated urticaria appears particularly common in infants and young children, often presenting with angioedema and systemic symptoms, and generally follows a favourable clinical course (5, 7, 8). However, the limited number of studies in adult populations, particularly outside the context of COVID-19, highlights an important gap in the literature and limits generalizability across age groups (7). Furthermore, available data do not permit direct comparison of long-term outcomes between SARS-CoV-2–associated urticaria and other viral infections in pediatric populations, as most studies did not stratify outcomes by specific viral pathogens, and the limited SARS-CoV-2–specific evidence in children further restricts such comparisons.

### Clinical implications

The findings of this review have direct relevance for clinical practice. Although infection-associated urticaria is typically self-limiting, clinicians should be aware that a subset of patients may develop persistent or chronic disease. Patients presenting with delayed-onset urticaria, particularly following SARS-CoV-2 infection, may warrant closer follow-up given the limited evidence suggesting an association with progression to chronic spontaneous urticaria. These findings support consideration of structured follow-up in patients with delayed-onset urticaria following viral infection, particularly in the context of SARS-CoV-2.

In pediatric populations, reassurance is appropriate in most cases; however, recurrence and chronicity remain clinically relevant and should be discussed with caregivers. Importantly, distinguishing infection-induced urticaria from drug-induced urticaria remains a key diagnostic challenge in routine practice.

These findings support a more individualized approach to follow-up and highlight the importance of clinician awareness of potential post-infectious disease trajectories.

#### Strengths and limitations

This review has several strengths. It was conducted in accordance with PRISMA 2020 guidelines and based on a prospectively registered protocol (11). A comprehensive search across multiple databases was performed, and study selection, data extraction, and risk of bias assessment were conducted using standardized methods. The focus on clinically relevant long-term outcomes provides important insight into the natural history of infection-associated urticaria beyond the acute phase.

However, several limitations should be considered. The small number of included studies and the marked clinical and methodological heterogeneity across populations, viral triggers, outcome definitions, and follow-up durations substantially limit the ability to draw firm or generalizable conclusions regarding the natural history of infection-associated urticaria. This reflects the limited number of studies specifically evaluating long-term outcomes of infection-associated urticaria and highlights an important evidence gap. Many studies included mixed-etiology cohorts without stratification by viral trigger, limiting causal inference (5, 6). Consequently, several reported long-term outcome estimates should be interpreted as indirect evidence relating to infection-associated urticaria rather than pathogen-specific viral risk estimates. Furthermore, evidence regarding delayed-onset urticaria and progression to chronicity is largely derived from SARS-CoV-2 cohorts, which may limit generalizability to other viral infections. Additionally, most studies were observational and at moderate risk of bias, with limited adjustment for confounding factors (4–8). The absence of standardized outcome definitions across studies further complicates comparison and synthesis of findings. A formal GRADE assessment was not performed due to heterogeneity in study designs and outcome measures, which limited comparability across studies.

### Key clinical messages

- Infection-associated urticaria is typically self-limiting
- Persistence beyond 6 months was reported in a minority of patients (∼5%–10%)
- Recurrence and chronicity were reported in up to one-third of patients in some mixed-etiology cohorts
- Delayed-onset urticaria, particularly following SARS-CoV-2 infection, may predict progression to chronic spontaneous urticaria
- Mixed aetiology in existing studies limits direct attribution of long-term outcomes specifically to viral triggers

## Conclusion

Infection-associated urticaria is generally an acute and self-limiting condition; however, a small but potentially clinically relevant subset of patients may experience prolonged or recurrent symptoms, including progression to chronic urticaria. Interpretation of the available evidence remains limited by mixed-etiology cohorts, inconsistent pathogen confirmation, and lack of pathogen-specific outcome stratification across studies.

These findings provide preliminary and hypothesis-generating insights into the long-term trajectory of infection-associated urticaria, with direct implications for patient counselling, follow-up, and risk stratification.

Current evidence is limited by small sample sizes, heterogeneous study designs, and lack of stratification according to infectious trigger. Further well-designed prospective studies with standardized outcome definitions and pathogen-specific analyses are needed to better characterize the long-term trajectory of infection-associated urticaria. Future studies should prioritize pathogen-confirmed cohorts and report long-term outcomes separately according to infectious aetiology to clarify the relationship between viral infection and long-term urticaria outcomes.

## Data Availability

The original contributions presented in the study are included in the article/Supplementary Material, further inquiries can be directed to the corresponding author.
