# Supplementary material for: Persistence, chronicity, and recurrence of infection-associated urticaria following viral infections in children and adults: a systematic review
Source: Front Allergy. 2026 May 26;7:1847423. doi: 10.3389/falgy.2026.1847423 (PMC13246635; doi:10.3389/falgy.2026.1847423)
Supplement: Supplementary file 1 [file Table1.docx]

Supplementary Table S1. Database-specific search syntax.

| **Scopus** |
| --- |
| S4 ( TITLE-ABS-KEY ( urticaria OR "chronic urticaria" OR "chronic spontaneous urticaria" OR hives ) AND TITLE-ABS-KEY ( "viral infection*" OR infection* OR virus* OR "post-viral" OR "post viral" OR "COVID-19" OR "SARS-CoV-2" ) AND TITLE-ABS-KEY ( persist* OR chronicity OR recurren* OR relaps* OR "long-term" OR "long term" OR outcome* ) ) (4,412)  S3 TITLE-ABS-KEY ( persist* OR chronicity OR recurren* OR relaps* OR "long-term" OR "long term" OR outcome* ) (9,402,408)  S2 TITLE-ABS-KEY ( "viral infection*" OR infection* OR virus* OR "post-viral" OR "post viral" OR "COVID-19" OR "SARS-CoV-2" ) (4,998,057)  S1 TITLE-ABS-KEY ( urticaria OR "chronic urticaria" OR "chronic spontaneous urticaria" OR hives ) (65,312) |
| **Embase <1974 to 2026 March 17>** |
| 1 exp urticaria/ or urticaria.ti,ab. or "chronic urticaria".ti,ab. or "chronic spontaneous urticaria".ti,ab. or hives.ti,ab. 66262  2 exp virus infection/ or exp coronavirus disease 2019/ or exp severe acute respiratory syndrome coronavirus 2/ or "viral infection*".ti,ab. or infection*.ti,ab. or virus*.ti,ab. or "post-viral".ti,ab. or "post viral".ti,ab. or "COVID-19".ti,ab. or "SARS-CoV-2".ti,ab. 4469980  3 (persist* or chronicity or recurren* or relaps* or "long-term" or "long term" or outcome*).ti,ab. 7500441  4 1 and 2 and 3 3435 |
| **CINHAL** |
| S4 S3 AND S2 AND S1 (114)  S3 (persist* OR chronicity OR recurren* OR relaps* OR "long-term" OR "long term" OR outcome*) (1,689,040)  S2 (MH "Virus Diseases+") OR (MH "Coronavirus Infections+") OR TI("viral infection*" OR infection* OR virus* OR "post-viral" OR "post viral" OR "COVID-19" OR "SARS-CoV-2") OR AB("viral infection*" OR infection* OR virus* OR "post-viral" OR "post viral" OR "COVID-19" OR "SARS-CoV-2") (604,460)  S1 (MH "Urticaria") OR TI(urticaria OR "chronic urticaria" OR "chronic spontaneous urticaria" OR hives) OR AB(urticaria OR "chronic urticaria" OR "chronic spontaneous urticaria" OR hives) (3,663) |
| **PubMed** |
| **4 Search:** ("Urticaria"[MeSH Terms] OR "Urticaria"[Title/Abstract] OR "chronic urticaria"[Title/Abstract] OR "chronic spontaneous urticaria"[Title/Abstract] OR "hives"[Title/Abstract]) AND ("Virus Diseases"[MeSH Terms] OR "viral infection*"[Title/Abstract] OR "infection*"[Title/Abstract] OR "virus*"[Title/Abstract] OR "post-viral"[Title/Abstract] OR "post-viral"[Title/Abstract] OR "COVID-19"[MeSH Terms] OR "COVID-19"[Title/Abstract] OR "SARS-CoV-2"[Title/Abstract]) AND ("persist*"[Title/Abstract] OR "chronicity"[Title/Abstract] OR "recurren*"[Title/Abstract] OR "relaps*"[Title/Abstract] OR "long-term"[Title/Abstract] OR "long-term"[Title/Abstract] OR "outcome*"[Title/Abstract]) (646)  **3 Search:** "persist*"[Title/Abstract] OR "chronicity"[Title/Abstract] OR "recurren*"[Title/Abstract] OR "relaps*"[Title/Abstract] OR "long-term"[Title/Abstract] OR "long term"[Title/Abstract] OR "outcome*"[Title/Abstract] (5,135,906)  **2 Search:** "Virus Diseases"[MeSH Terms] OR "viral infection*"[Title/Abstract] OR "infection*"[Title/Abstract] OR "virus*"[Title/Abstract] OR "post-viral"[Title/Abstract] OR "post-viral"[Title/Abstract] OR "COVID-19"[MeSH Terms] OR "COVID-19"[Title/Abstract] OR "SARS-CoV-2"[Title/Abstract] (3,318,412)  **1 Search:** "Urticaria"[MeSH Terms] OR "Urticaria"[Title/Abstract] OR "chronic urticaria"[Title/Abstract] OR "chronic spontaneous urticaria"[Title/Abstract] OR "hives"[Title/Abstract] (30,038) |
| **Web of Science Core Collection** |
| S4 urticaria OR "chronic urticaria" OR "chronic spontaneous urticaria" OR hives (Topic) and "viral infection*" OR infection* OR virus* OR "post-viral" OR "post viral" OR "COVID-19" OR "SARS-CoV-2" (Topic) and persist* OR chronicity OR recurren* OR relaps* OR "long-term" OR "long term" OR outcome* (Topic) (577)  S3 persist* OR chronicity OR recurren* OR relaps* OR "long-term" OR "long term" OR outcome* (Topic) (7,406,952)  S2 "viral infection*" OR infection* OR virus* OR "post-viral" OR "post viral" OR "COVID-19" OR "SARS-CoV-2" (Topic) (3,573,930)  S1 urticaria OR "chronic urticaria" OR "chronic spontaneous urticaria" OR hives (Topic) (32,158) |
