# Supplementary material for: Persistence, chronicity, and recurrence of infection-associated urticaria following viral infections in children and adults: a systematic review
Source: Front Allergy. 2026 May 26;7:1847423. doi: 10.3389/falgy.2026.1847423 (PMC13246635; doi:10.3389/falgy.2026.1847423)
Supplement: Supplementary file 2 [file Table2.docx]

Supplementary Table S2. Studies excluded at full-text stage.

| Article excluded | Reason for exclusion |
| --- | --- |
| Abdou AG, Elshayeb EI, Farag AGA, Elnaidany NF. Helicobacter pylori infection in patients with chronic urticaria: correlation with pathologic findings in gastric biopsies. Int J Dermatol. 2009;48:464–469. | Evaluated the association between Helicobacter pylori infection and pre-existing chronic urticaria, focusing on symptom severity and response to eradication therapy. The study did not assess urticaria occurring after viral infection or report long-term outcomes such as persistence, chronicity, recurrence, or relapse following infection, and therefore was not aligned with the study objective focusing on post-viral urticaria trajectories. |
| AlBalbeesi A, Alsalman H, Alotaibi H, et al. Prevalence of Helicobacter pylori Infection Among Rosacea and Chronic Spontaneous Urticaria Patients in a Tertiary Hospital in Riyadh, Saudi Arabia. Cureus. 2021;13:e17617. | Cross-sectional study evaluating the prevalence and association of Helicobacter pylori infection among patients with pre-existing chronic spontaneous urticaria, without longitudinal follow-up or primary outcome assessment beyond baseline. Did not assess urticaria occurring after viral infection or report long-term outcomes such as persistence, chronicity, recurrence, or relapse, and therefore was not aligned with the study objective focusing on post-viral urticaria trajectories. |
| Aleksić J, Aleksić N, Stojanović N, et al. Evaluation of etiological factors and clinical parameters of acute urticaria in children treated at a pediatric internal diseases clinic. MedPodml. 2019;70:43–47. | Retrospective study evaluating etiological factors and clinical characteristics of acute urticaria in children. Although infections were identified as common preceding factors, the study focused exclusively on acute presentations without longitudinal follow-up. It did not assess long-term outcomes such as persistence, chronicity, recurrence, or relapse following viral infection, and therefore was not aligned with the study objective focusing on post-viral urticaria trajectories. |
| Ali S, Ghazanfar MN, Holm JG, Thomsen SF. Events during the 3 Months Immediately Preceding Onset of Chronic Urticaria: A Questionnaire Study. Dermatology. 2021;237:320–322. | Questionnaire-based study evaluating events preceding the onset of chronic urticaria in patients with pre-existing disease, without specific assessment of viral infection as the primary exposure or longitudinal follow-up beyond baseline. Did not evaluate urticaria occurring after viral infection or report long-term outcomes such as persistence, chronicity, recurrence, or relapse, and therefore was not aligned with the study objective focusing on post-viral urticaria trajectories. |
| Ari H, Guvenir H, Toyran M, et al. Etiological Assessment of Acute Urticaria in Children. Turkish J Pediatr Dis. 2023;17:13–18. | Retrospective study evaluating the etiology and triggers of acute urticaria in children, including infection-associated cases, without longitudinal follow-up or primary outcome assessment beyond the initial episode. Outcomes were limited to identification of triggers and recurrence of acute urticaria episodes, without evaluation of persistence, chronicity, or relapse following infection, and therefore was not aligned with the study objective focusing on post-viral urticaria trajectories. |
| Ben-Shoshan M, Kanani A, Kalicinsky C, Watson W. Urticaria. Allergy Asthma Clin Immunol. 2024;20(Suppl 3):64. | Narrative review summarizing the classification, etiology, diagnosis, and management of urticaria without providing primary data or study-level outcomes. Did not evaluate or report long-term outcomes such as persistence, chronicity, recurrence, or relapse of urticaria following viral infection. |
| Bezirganoglu H, Arik Yilmaz E, Sahiner UM, et al. The common triggers of urticaria in children admitted to the pediatric emergency room. Pediatr Dermatol. 2022;39:695–701. | Retrospective study evaluating triggers of urticaria in children presenting to the emergency department, without longitudinal follow-up or primary outcome assessment beyond the acute episode. Did not evaluate urticaria occurring after viral infection or report long-term outcomes such as persistence, chronicity, recurrence, or relapse, and therefore was not aligned with the study objective focusing on post-viral urticaria trajectories. |
| Bostan E, Zaid F, Karaduman A, et al. The effect of COVID-19 on patients with chronic spontaneous urticaria treated with omalizumab and antihistamines: A cross-sectional, comparative study. J Cosmet Dermatol. 2021;20:3369–3375. | Cross-sectional study evaluating the prevalence and clinical impact of COVID-19 infection in patients with pre-existing chronic spontaneous urticaria, without longitudinal follow-up or primary outcome assessment beyond the study period. Did not assess urticaria occurring after viral infection or report long-term outcomes such as persistence, chronicity, recurrence, or relapse, and therefore was not aligned with the study objective focusing on post-viral urticaria trajectories. |
| Brailo V, Vidović Juras D, Stanimirović A, et al. Dental infection and dermatological diseases: analysis of ninety-two patients and review of the literature. Acta Clin Croat. 2015;54:77–82. | Retrospective study evaluating the association between dental (bacterial) infection and various dermatological diseases, including a subset of urticaria cases, without longitudinal follow-up focused on urticaria outcomes or assessment beyond treatment response. Did not assess urticaria occurring after viral infection or report long-term outcomes such as persistence, chronicity, recurrence, or relapse, and therefore was not aligned with the study objective focusing on post-viral urticaria trajectories. |
| Buono EV, Giannì G, Scavone S, Caffarelli C. Clinical characteristics, investigations and treatment in children with chronic urticaria: an observational study. Medicina. 2024;60:704. | Observational study evaluating clinical characteristics, comorbidities, and treatment outcomes in children with pre-existing chronic urticaria. Although follow-up data and remission rates were reported, the study did not assess urticaria occurring after viral infection, and infections evaluated were not identified as causal triggers of urticaria or linked to long-term outcomes. Therefore, it was not aligned with the study objective focusing on post-viral urticaria trajectories. |
| Burlando M, Russo R, Cozzani E, Parodi A. Six months into the pandemic. A review of skin manifestations in SARS-CoV-2 infection. Dermatol Ther. 2021;34:e14641. | Review article summarizing cutaneous manifestations of COVID-19, including urticarial eruptions, without primary patient-level data or longitudinal follow-up beyond the initial illness. Did not evaluate or report long-term outcomes such as persistence, chronicity, recurrence, or relapse of urticaria following viral infection, and therefore was not aligned with the study objective focusing on post-viral urticaria trajectories. |
| Buss YA, Garrelfs UC, Sticherling M. Chronic urticaria – which clinical parameters are pathogenetically relevant? A retrospective investigation of 339 patients. J Dtsch Dermatol Ges. 2007;5:22–29. | Retrospective study evaluating clinical characteristics and potential triggers in patients with predominantly chronic urticaria, without longitudinal follow-up or primary outcome assessment beyond baseline evaluation. Did not assess urticaria occurring after viral infection or report long-term outcomes such as persistence, chronicity, recurrence, or relapse following infection, and therefore was not aligned with the study objective focusing on post-viral urticaria trajectories. |
| Buttgereit T, Vera C, Aulenbacher F, et al. Patients with chronic spontaneous urticaria who have wheals, angioedema, or both, differ demographically, clinically, and in response to treatment—results from CURE. J Allergy Clin Immunol Pract. 2023;11:3515–3525.e4. | Retrospective analysis of registry data evaluating clinical characteristics, comorbidities, and treatment response in patients with pre-existing chronic spontaneous urticaria. The study did not assess urticaria occurring after viral infection and did not evaluate long-term outcomes such as persistence, chronicity, recurrence, or relapse following viral infection, and therefore was not aligned with the study objective focusing on post-viral urticaria trajectories. |
| Calado G, Loureiro G, Machado D, et al. Streptococcal tonsillitis as a cause of urticaria. Allergol Immunopathol (Madr). 2012;40:341–345. | Retrospective study evaluating the association between streptococcal tonsillitis and chronic or recurrent urticaria in a small, highly selected cohort, without focus on viral infection as the exposure of interest. Although clinical outcomes such as resolution and persistence were reported, the study did not assess urticaria occurring after viral infection or report long-term outcomes in a viral context, and therefore was not aligned with the study objective focusing on post-viral urticaria trajectories. |
| Çetinkaya F, Uysalol EP, Aylan Gelen S, Besbenli K. Etiological evaluation of acute urticaria in children admitted to an inner city hospital of Turkey. Asthma Allergy Immunol. 2011;9:15–19. | Prospective study evaluating etiological factors in children presenting with acute urticaria in an emergency setting. Although infections were identified as potential triggers, the study focused exclusively on acute presentations without longitudinal follow-up. It did not assess long-term outcomes such as persistence, chronicity, recurrence, or relapse of urticaria following viral infection, and therefore was not aligned with the study objective focusing on post-viral urticaria trajectories. |
| Chansakulporn S, Pongpreuksa S, Sangacharoenkit P, Pacharn P, Visitsunthorn N, Vichyanond P, Jirapongsananuruk O. The natural history of chronic urticaria in childhood: a prospective study. J Am Acad Dermatol. 2014;71:663–668. | Prospective study evaluating the natural course and remission of chronic urticaria in children with mixed and predominantly idiopathic aetiologies. Although long-term outcomes such as remission rates were assessed, viral infection was not defined as the primary exposure and no infection-specific subgroup analysis was performed. The study did not evaluate urticaria occurring after viral infection or report long-term outcomes specific to infection-related cases, and therefore was not aligned with the study objective focusing on post-viral urticaria trajectories. |
| Chen CM, Huang WT, Chang LJ, et al. Peptic ulcer disease is associated with increased risk of chronic urticaria independent of Helicobacter pylori infection: a population-based cohort study. Am J Clin Dermatol. 2021;22:129–137. | Population-based cohort study evaluating the risk of developing chronic urticaria in patients with peptic ulcer disease, rather than urticaria occurring after viral infection. Although longitudinal outcomes were assessed, the study focused on incident risk rather than post-infectious urticaria trajectories and did not evaluate persistence, chronicity, recurrence, or relapse following viral infection, and therefore was not aligned with the study objective focusing on post-viral urticaria trajectories. |
| Comert S, Celebioglu E, Karakaya G, Kalyoncu AF. The general characteristics of acute urticaria attacks and the factors predictive of progression to chronic urticaria. Allergol Immunopathol (Madr). 2013;41:239–245. | Observational study evaluating triggers and clinical characteristics of acute urticaria and comparing these with a separate chronic urticaria cohort to identify potential predictive factors. Although infections were reported as possible triggers, the study did not follow a defined cohort longitudinally to assess post-infectious outcomes. It did not evaluate persistence, chronicity, recurrence, or relapse following viral infection, and therefore was not aligned with the study objective focusing on post-viral urticaria trajectories. |
| Cordeiro D, Palhas P, Souza P, et al. Diagnostic profile among patients with chronic urticaria/angioedema attending a reference clinic in Brazil: the role of auto-immunity. World Allergy Organ J. 2015;8(Suppl 1):A104. | Evaluated the diagnostic and immunological profile of patients with pre-existing chronic urticaria/angioedema, focusing on potential triggers and autoimmune features, without assessment of viral infection as the exposure of interest or longitudinal outcomes following infection. The study did not assess urticaria occurring after viral infection or report long-term outcomes such as persistence, chronicity, recurrence, or relapse, and therefore was not aligned with the study objective focusing on post-viral urticaria trajectories. |
| Craffert V, Day C, Peter J. New-onset chronic spontaneous urticaria post–COVID-19 vaccination—South African case series. J Allergy Clin Immunol Glob. 2023;2:100154. | Evaluated new-onset chronic spontaneous urticaria following COVID-19 vaccination in a case series, rather than urticaria associated with viral infection. Although long-term outcomes were reported, the exposure of interest was vaccine-related rather than infection-related, and therefore not aligned with the study objective focusing on post-viral urticaria trajectories. |
| Cribier BJ, Santinelli F, Schmitt C, et al. Chronic urticaria is not significantly associated with hepatitis C or hepatitis G infection: a case-control study. Arch Dermatol. 1999;135:1335–1339. | Evaluated the association between hepatitis C and hepatitis G viral infections and pre-existing chronic urticaria using a case-control design, focusing on potential etiological links rather than disease progression. The study did not assess urticaria occurring after viral infection or report long-term outcomes such as persistence, chronicity, recurrence, or relapse following infection, and therefore was not aligned with the study objective focusing on post-viral urticaria trajectories. |
| Cuevas Acuña MT, López García AI, Paz Martínez D, et al. Frecuencia de infección por Helicobacter pylori en pacientes con urticaria crónica del Hospital Universitario de Puebla. Rev Alerg Mex. 2006;53:174–178. | valuated the frequency of Helicobacter pylori infection in patients with pre-existing chronic urticaria using a cross-sectional design, focusing on potential etiological associations rather than disease progression. The study did not assess urticaria occurring after viral infection or report long-term outcomes such as persistence, chronicity, recurrence, or relapse following infection, and therefore was not aligned with the study objective focusing on post-viral urticaria trajectories. |
| da Costa Farinha IF, Pereira HSA, Lemos SCG, et al. Hospital admissions for urticaria in a pediatric emergency department of a tertiary care hospital. Allergol Immunopathol (Madr). 2023;51:117–123. | Retrospective observational study evaluating clinical characteristics and suspected triggers of urticaria in a pediatric emergency department setting. Although infections were identified as common suspected triggers, the study primarily included acute urticaria cases and did not assess long-term outcomes or post-infectious disease trajectories. Chronic urticaria cases were not linked to viral infection. Therefore, the study was not aligned with the objective focusing on post-viral urticaria trajectories. |
| Elhendawy M, Hagras MM, Soliman SS, Shaker ESE. Positive effect of Helicobacter pylori treatment on outcome of patients with chronic spontaneous urticaria: a randomized double-blind pilot study. Am J Clin Pathol. 2021;155:405–411. | Evaluated the association between Helicobacter pylori infection and pre-existing chronic spontaneous urticaria and assessed the effect of bacterial eradication therapy on clinical outcomes in a randomized controlled trial. The study did not assess urticaria occurring after viral infection or report long-term outcomes such as persistence, chronicity, recurrence, or relapse following infection, and therefore was not aligned with the study objective focusing on post-viral urticaria trajectories. |
| Federman DG, Kirsner RS, Moriarty JP, Concato J. The effect of antibiotic therapy for patients infected with Helicobacter pylori who have chronic urticaria. J Am Acad Dermatol. 2003;49:861–864. | Systematic review summarizing existing studies evaluating the effect of Helicobacter pylori eradication therapy on patients with pre-existing chronic urticaria, without providing primary data or study-level outcomes. The review did not assess urticaria occurring after viral infection or report long-term outcomes such as persistence, chronicity, recurrence, or relapse following infection, and therefore was not aligned with the study objective focusing on post-viral urticaria trajectories. |
| Floridia M, Pagnanelli G, Piccinni G, Weimer LE, Onder G, et al. Persisting or recurrent dermatological manifestations in Long-COVID: data from a national cohort of 1741 patients from Italy. J Am Acad Dermatol. 2026. | Observational cohort study evaluating a broad range of persistent or recurrent dermatological manifestations following COVID-19 infection. Although a small subset of patients presented with urticaria, the study included heterogeneous skin conditions and did not focus specifically on urticaria as a primary outcome. While outcomes were assessed beyond the acute phase, the study did not provide stratified analysis or extractable long-term outcomes specifically for urticaria following viral infection. It did not assess post-infectious trajectories such as persistence, chronicity, recurrence, or relapse in a defined urticaria cohort, and therefore was not aligned with the study objective focusing on post-viral urticaria trajectories. |
| Forrer A, Khieu V, Schär F, et al. Strongyloides stercoralis is associated with significant morbidity in rural Cambodia, including stunting in children. PLoS Negl Trop Dis. 2017;11:e0005685. | Evaluated the association between Strongyloides stercoralis infection and clinical symptoms, including urticaria, in a cross-sectional and before–after treatment study, where urticaria was reported as a manifestation of parasitic infection rather than a primary outcome. The study did not assess urticaria occurring after viral infection or report long-term outcomes such as persistence, chronicity, recurrence, or relapse, and therefore was not aligned with the study objective focusing on post-viral urticaria trajectories. |
| Freeman EE, McMahon DE, Lipoff JB, et al. The spectrum of COVID-19–associated dermatologic manifestations: An international registry of 716 patients from 31 countries. J Am Acad Dermatol. 2020;83:1118–1129. | Described a broad spectrum of acute dermatologic manifestations of COVID-19, including urticarial eruptions, within an international registry-based case series, without evaluating long-term outcomes such as persistence, chronicity, recurrence, or relapse of urticaria. Urticaria was not analysed as a primary condition, follow-up duration was insufficient to assess chronic urticaria (≥6 weeks), and no outcome-specific longitudinal analysis relevant to post-viral urticaria trajectories was provided. |
| Fukuda S, Shimoyama T, Umegaki N, et al. Effect of Helicobacter pylori eradication in the treatment of Japanese patients with chronic idiopathic urticaria. J Gastroenterol. 2004;39:827–830. | Evaluated the association between Helicobacter pylori infection and pre-existing chronic idiopathic urticaria and assessed the effect of bacterial eradication therapy on clinical outcomes. The study did not assess urticaria occurring after viral infection or report long-term outcomes such as persistence, chronicity, recurrence, or relapse following infection, and therefore was not aligned with the study objective focusing on post-viral urticaria trajectories. |
| Galván Casas C, Català A, Carretero Hernández G, et al. Classification of the cutaneous manifestations of COVID-19: a rapid prospective nationwide consensus study in Spain with 375 cases. Br J Dermatol. 2020;183:71–77. | Cross-sectional study describing acute cutaneous manifestations of COVID-19, including urticarial lesions, without longitudinal follow-up or primary outcome assessment beyond the initial illness. Did not evaluate or report long-term outcomes such as persistence, chronicity, recurrence, or relapse of urticaria following viral infection. |
| Godse KV, Zawar V. Chronic urticaria associated with tinea infection and success with antifungal therapy—a report of four cases. Int J Infect Dis. 2010;14S:e364–e365. | Case series describing four patients with chronic urticaria associated with dermatophyte (tinea) infection, where urticaria was considered a secondary reaction to fungal infection and resolved following antifungal therapy. The study did not assess urticaria occurring after viral infection or report long-term outcomes such as persistence, chronicity, recurrence, or relapse, and therefore was not aligned with the study objective focusing on post-viral urticaria trajectories. |
| Gold-Olufadi S, Ayanlowo O, Akinkugbe AO, Otrofanowei E. Clinical and aetiologic profile of patients with chronic urticaria at the outpatient clinic of a tertiary hospital in Lagos, Nigeria: a cross-sectional observational study. Pan Afr Med J. 2021;40:141. | Cross-sectional observational study describing the clinical profile and potential etiological factors in patients with pre-existing chronic urticaria, without assessment of temporal relationships between infection and disease onset or longitudinal outcomes. The study did not evaluate urticaria occurring after viral infection or report long-term outcomes such as persistence, chronicity, recurrence, or relapse following infection, and therefore was not aligned with the study objective focusing on post-viral urticaria trajectories. |
| Hatzenbuehler LA, Tobin-D’Angelo M, Drenzek C, et al. Pediatric Dental Clinic–Associated Outbreak of Mycobacterium abscessus Infection. J Pediatric Infect Dis Soc. 2017;6:e116–e122. | Outbreak investigation of Mycobacterium abscessus infections following dental procedures, focusing on clinical presentation, management, and complications of odontogenic infection. Urticaria was reported only as an adverse reaction to antibiotic therapy in a small subset of patients and was not evaluated as a primary outcome. The study did not assess urticaria occurring after viral infection or report long-term outcomes such as persistence, chronicity, recurrence, or relapse of urticaria, and therefore was not aligned with the study objective focusing on post-viral urticaria trajectories. |
| Hellmig S, Troch K, Ott SJ, Fölsch UR, Schwarz T. Yersinia enterocolitica: another factor in the pathogenesis of chronic urticaria? Clin Exp Dermatol. 2009;34:e292. | Correspondence reporting a retrospective analysis of patients with pre-existing chronic urticaria, evaluating the association between bacterial infections (e.g., Yersinia enterocolitica and Helicobacter pylori) and disease duration. The study did not assess urticaria occurring after viral infection and focused on potential triggers and disease associations rather than post-infectious disease trajectories. It did not report long-term outcomes such as persistence, chronicity, recurrence, or relapse following viral infection, and therefore was not aligned with the study objective focusing on post-viral urticaria trajectories. |
| Hellmig S, Troch K, Ott SJ, Schwarz T, Fölsch UR. Role of Helicobacter pylori Infection in the Treatment and Outcome of Chronic Urticaria. Helicobacter. 2008;13:341–345. | Observational study evaluating the impact of Helicobacter pylori infection and eradication therapy on the clinical course of patients with pre-existing chronic urticaria. Although long-term follow-up was reported, the study focused on treatment response and disease course in established urticaria rather than urticaria occurring after viral infection. It did not assess post-infectious onset or report outcomes such as persistence, chronicity, recurrence, or relapse following viral infection, and therefore was not aligned with the study objective focusing on post-viral urticaria trajectories. |
| Kan SY, Koh MJA, Wee LWY. Urticaria multiforme in Asian children. J Paediatr Child Health. 2024;60:538–543. | Retrospective case series describing clinical features, triggers, and outcomes of urticaria multiforme, a subtype of acute urticaria, in children. Although viral infections were identified as common triggers, the study focused on acute, self-limiting presentations with symptom resolution within weeks and did not include longitudinal follow-up beyond the acute phase. It did not assess long-term outcomes such as persistence, chronicity, recurrence, or relapse of urticaria following viral infection, and therefore was not aligned with the study objective focusing on post-viral urticaria trajectories. |
| Kasperska-Zając A, Brzoza Z, Rogala B. Plasma concentration of platelet factor 4 in patients with acute urticaria in the course of acute respiratory tract infection. Adv Clin Exp Med. 2006;15:995–998. | Experimental study evaluating platelet activation in a small cohort of patients with acute urticaria associated with respiratory tract infection. Although infection-related urticaria was assessed, the study focused on acute-phase pathophysiological mechanisms without longitudinal follow-up. It did not evaluate long-term outcomes such as persistence, chronicity, recurrence, or relapse, and therefore was not aligned with the study objective focusing on post-viral urticaria trajectories. |
| Kauppinen K, Juntunen K, Lanki H. Urticaria in Children: Retrospective Evaluation and Follow-Up. Allergy. 1984;39:469–472. | Retrospective study evaluating the etiology and long-term outcomes of urticaria in children with mixed triggers, including physical factors, foods, drugs, and a minority of infections. Although follow-up data were reported, outcomes were not stratified by specific aetiologies and viral infections were not defined as the primary exposure. The study did not specifically assess urticaria occurring after viral infection or report long-term outcomes attributable to infection-related cases, and therefore was not aligned with the study objective focusing on post-viral urticaria trajectories. |
| Kennedy JL, Stallings AP, Platts-Mills TAE, et al. Galactose-α-1,3-galactose and Delayed Anaphylaxis, Angioedema, and Urticaria in Children. Pediatrics. 2013;131:e1545–e1552. | Observational study evaluating delayed urticaria, angioedema, and anaphylaxis in children associated with IgE-mediated allergy to galactose-α-1,3-galactose (α-Gal) following consumption of mammalian meat. The study focuses on food allergy–related urticaria rather than infection-related disease and does not assess urticaria occurring after viral infection. It did not evaluate long-term outcomes such as persistence, chronicity, recurrence, or relapse in a post-infectious context, and therefore was not aligned with the study objective focusing on post-viral urticaria trajectories. |
| Khakoo G, Sofianou-Katsoulis A, Perkin MR, Lack G. Clinical features and natural history of physical urticaria in children. Pediatr Allergy Immunol. 2008;19:363–366. | Retrospective study evaluating the clinical features and natural history of physical urticaria in children, a subtype of chronic inducible urticaria triggered by physical stimuli such as pressure, temperature, or exercise. The study population consisted of patients with pre-existing chronic urticaria of non-infectious etiology and did not assess urticaria occurring after viral infection. It did not evaluate post-infectious outcomes or report persistence, chronicity, recurrence, or relapse in a viral context, and therefore was not aligned with the study objective focusing on post-viral urticaria trajectories. |
| Khan S. Urticaria in patients with diabetes: Adverse drug reaction or relapse of underlying autoimmune urticaria? Indian J Med Res. 2019;149:423–425. | Retrospective study evaluating urticaria in patients with diabetes, focusing on adverse drug reactions, autoimmune associations, and comorbid conditions. Urticaria cases were primarily attributed to medications, metabolic factors, or autoimmune disease, with only a small number of infection-related cases that were not viral-specific and not analyzed separately. The study did not assess urticaria occurring after viral infection or report long-term outcomes such as persistence, chronicity, recurrence, or relapse in a post-infectious context, and therefore was not aligned with the study objective focusing on post-viral urticaria trajectories. |
| Kim MH. Epidemiological insights into chronic urticaria, vitiligo, alopecia areata, and herpes zoster following COVID-19 infection: A nationwide population-based study. J Dermatol. 2025;52:499–504. | Evaluated the incidence and risk of new-onset chronic urticaria following COVID-19 infection in a large population-based cohort, rather than the long-term clinical course of urticaria. Although the exposure of interest was infection-related, the study focused on disease occurrence and did not report long-term outcomes such as persistence, chronicity, recurrence, or relapse, and therefore was not aligned with the study objective focusing on post-viral urticaria trajectories. |
| Kim H, Hyun MC, Choi BS. Natural history and influencing factors of chronic urticaria in children. Allergy Asthma Immunol Res. 2022;14:73–84. | Retrospective study evaluating the natural course and prognostic factors of chronic urticaria in children with predominantly idiopathic or mixed aetiologies. Although long-term outcomes such as remission rates were reported, viral infection was not defined as the primary exposure and was only identified in a small number of cases without subgroup analysis. The study did not assess urticaria occurring after viral infection or evaluate long-term outcomes specific to infection-related cases, and therefore was not aligned with the study objective focusing on post-viral urticaria trajectories. |
| Kocatürk E, Muñoz M, Elieh-Ali-Komi D, et al. How Infection and Vaccination Are Linked to Acute and Chronic Urticaria: A Special Focus on COVID-19. Viruses. 2023;15:1585. | Narrative review summarizing the role of viral infections and vaccination in acute and chronic urticaria, without providing primary data or study-level outcomes. Did not report extractable long-term outcomes such as persistence, chronicity, recurrence, or relapse of urticaria following viral infection at the individual study level. |
| Kolkhir P, Bonnekoh H, Metz M, Maurer M. Chronic spontaneous urticaria: a review. JAMA. 2024;332(17):1464–1477. | Narrative review summarizing the epidemiology, pathophysiology, diagnosis, and management of chronic spontaneous urticaria without providing primary data or study-level outcomes. Did not specifically evaluate or report long-term outcomes such as persistence, chronicity, recurrence, or relapse of urticaria following viral infection. |
| Kolkhir P, Giménez-Arnau AM, Kulthanan K, et al. Urticaria. Nat Rev Dis Primers. 2022;8:61. | Narrative review summarizing the epidemiology, pathophysiology, diagnosis, and management of urticaria without providing primary patient-level data or study-specific outcomes. Although infections, including viral infections, are discussed as potential triggers, the article does not evaluate urticaria occurring after viral infection in defined cohorts or report extractable long-term outcomes such as persistence, chronicity, recurrence, or relapse. Therefore, it was not aligned with the study objective focusing on post-viral urticaria trajectories. |
| Koumaki D, Koumaki V, Boumpoucheropoulos S, et al. Childhood acute urticaria and seasonal patterns presenting in the emergency department of a teaching hospital in London, United Kingdom. Pediatr Emerg Care. 2022;38:e385–e386. | Retrospective descriptive study evaluating the clinical characteristics, triggers, and seasonality of acute urticaria in children presenting to the emergency department. Although infections were identified as potential triggers, the study focused exclusively on acute presentations without longitudinal follow-up. It did not assess long-term outcomes such as persistence, chronicity, recurrence, or relapse of urticaria following viral infection, and therefore was not aligned with the study objective focusing on post-viral urticaria trajectories. |
| Konstantinou GN, Papadopoulos NG, Tavladaki T, et al. Childhood acute urticaria in northern and southern Europe shows a similar epidemiological pattern and significant meteorological influences. Pediatr Allergy Immunol. 2011;22:36–42. | Retrospective epidemiological study evaluating the incidence, potential triggers, and environmental influences of acute urticaria in children presenting to emergency departments. Although infections, particularly respiratory infections, were frequently reported as associated triggers, the study did not include longitudinal follow-up or assess long-term outcomes such as persistence, chronicity, recurrence, or relapse. Therefore, it was not aligned with the study objective focusing on post-viral urticaria trajectories. |
| Kozel MMA, Mekkes JR, Bossuyt PMM, Bos JD. The Effectiveness of a History-Based Diagnostic Approach in Chronic Urticaria and Angioedema. Arch Dermatol. 1998;134:1575–1580. | Prospective study evaluating diagnostic strategies in patients with chronic urticaria and/or angioedema, focusing on identification of underlying causes through history taking and laboratory investigations. The study included patients with established chronic urticaria of mixed aetiologies and did not specifically assess urticaria occurring after viral infection. Outcomes were related to diagnostic yield rather than long-term disease trajectories, and no extractable data on persistence, chronicity, recurrence, or relapse following viral infection were reported. Therefore, it was not aligned with the study objective focusing on post-viral urticaria trajectories. |
| Kulthanan K, Cheepsomsong M, Jiamton S. Urticarial Vasculitis: Etiologies and Clinical Course. Asian Pac J Allergy Immunol. 2009;27:95–102. | Retrospective study evaluating aetiologies and clinical outcomes of urticarial vasculitis, a distinct clinicopathological entity characterized by lesions lasting >24 hours with histologic evidence of leukocytoclastic vasculitis. The study population did not represent urticaria as defined in the inclusion criteria and included mixed aetiologies, with infections not limited to viral causes and not analyzed separately. It did not assess urticaria occurring after viral infection or report post-viral long-term outcomes such as persistence, chronicity, recurrence, or relapse. Therefore, it was not aligned with the study objective focusing on post-viral urticaria trajectories. |
| Kulthanan K, Chiawsirikajorn Y, Jiamton S. Acute Urticaria: Etiologies, Clinical Course and Quality of Life. Asian Pac J Allergy Immunol. 2008;26:1–9. | Prospective study evaluating aetiologies, clinical features, and short-term outcomes of acute urticaria. Although infections were identified as common triggers, the study focused on the acute phase with rapid symptom resolution in most patients and did not include follow-up beyond the initial illness. It did not assess long-term outcomes such as persistence, chronicity, recurrence, or relapse of urticaria following viral infection, and therefore was not aligned with the study objective focusing on post-viral urticaria trajectories. |
| Kumar P, Radha G, Muthukrishnan M, Chandrasekaran B, Subbiah P, Raman J. Cutaneous manifestations associated with COVID-19 infection in a COVID-designated hospital in North Chennai: A descriptive cross-sectional study. Indian Dermatol Online J. 2023;14:67–71. | Descriptive cross-sectional study evaluating cutaneous manifestations, including urticarial eruptions, in patients with COVID-19 infection without longitudinal follow-up. Outcomes were limited to acute, transient skin findings and did not assess long-term outcomes such as persistence, chronicity, recurrence, or relapse of urticaria. |
| Kumaran MS, Mahajan R, Goyal N, Parsad D. Clinico-epidemiological features of chronic urticaria in children: A retrospective analysis of 296 children from a tertiary care institute in Northern India. Indian J Dermatol Venereol Leprol. 2020;86:50–54. | Retrospective study evaluating the clinical and epidemiological profile of children with established chronic urticaria of mixed aetiologies. Although infections were reported as potential precipitating factors, they were not limited to viral causes and were not analyzed separately. The study did not assess urticaria occurring after viral infection or report infection-specific long-term outcomes such as persistence, chronicity, recurrence, or relapse. Therefore, it was not aligned with the study objective focusing on post-viral urticaria trajectories. |
| Lascialfari G, Sarti L, Barni S, Liccioli G, Paladini E, Guarnieri V, et al. Relapse or worsening of chronic spontaneous urticaria during SARS-CoV-2 infection and vaccination in children: A telemedicine follow-up. Allergol Immunopathol (Madr). 2022;50(SP2):1–7. | Evaluated relapse or worsening of pre-existing chronic spontaneous urticaria in children during SARS-CoV-2 infection and vaccination, rather than urticaria occurring after viral infection. Although outcomes such as relapse and disease worsening were reported, the study population consisted of patients with established urticaria, and therefore was not aligned with the study objective focusing on post-viral urticaria trajectories. |
| Lee S, Park J, Kang J, Smith L, Rahmati M, Lee H, Yon DK. Risks of chronic urticaria after SARS-CoV-2 infection: binational population-based cohort studies from South Korea and Japan. J Allergy Clin Immunol Pract. 2024;12:2540–2542.e3. | Population-based cohort study evaluating the risk of incident chronic urticaria following SARS-CoV-2 infection, rather than the clinical course of urticaria after viral infection. Although longitudinal data were included, the study focused on disease occurrence and did not assess long-term outcomes such as persistence, chronicity, recurrence, or relapse of urticaria following infection, and therefore was not aligned with the study objective focusing on post-viral urticaria trajectories. |
| Legrain V, Taieb A, Sage T, Maleville J. Urticaria in Infants: A Study of Forty Patients. Pediatr Dermatol. 1990;7:101–107. | Retrospective study evaluating clinical features, aetiologies, and outcomes of urticaria in infants, predominantly involving acute urticaria with a small proportion of recurrent and chronic cases. Although infections, including viral infections, were identified as common triggers, the study focused mainly on the acute phase and did not provide stratified analysis or extractable long-term outcomes specifically following viral infection. It did not assess post-infectious trajectories such as persistence, chronicity, recurrence, or relapse in a defined viral cohort, and therefore was not aligned with the study objective focusing on post-viral urticaria trajectories. |
| Li CX, Li HG, Zhang BB, Huang Y, Wang BM, Shen YH, Wang XX, Yang WQ, Gu Y, Guo YF, Zhang H. Natural history and prognostic factors of chronic urticaria in children aged < 2 years: a single-centered, real-world, and retrospective study. Dermatol Ther. 2025;2025:1564070. | Retrospective study evaluating the natural history and prognostic factors of pre-existing chronic urticaria in young children. Although follow-up data and remission rates were reported, viral infections were not defined as the primary exposure and were only identified as nonspecific triggers without subgroup analysis. The study did not evaluate urticaria occurring after viral infection or assess long-term outcomes specific to infection-related cases, and therefore was not aligned with the study objective focusing on post-viral urticaria trajectories. |
| Lin YR, Liu TH, Wu TK, et al. Predictive factors of the duration of a first-attack acute urticaria in children. Am J Emerg Med. 2011;29:883–889. | Observational study evaluating predictors of short-term duration of acute urticaria in children. Although infections were identified as common aetiologies, the study focused on acute-phase disease with follow-up limited to days. It did not assess long-term outcomes such as persistence, chronicity, recurrence, or relapse following viral infection, and therefore was not aligned with the study objective focusing on post-viral urticaria trajectories. |
| Liu T-H, Lin Y-R, Yang K-C, Tsai Y-G, Fu Y-C, Wu T-K, Wu H-P. Significant factors associated with severity and outcome of an initial episode of acute urticaria in children. Pediatr Allergy Immunol. 2010;21:1043–1051. | Retrospective study evaluating predictors of severity and short-term outcomes of an initial episode of acute urticaria in children. Although infections were identified as common triggers, including presumed viral infections, the study focused exclusively on the acute phase with follow-up limited to symptom resolution within days to weeks. It did not provide stratified analysis or extractable long-term outcomes specifically following viral infection. It did not assess post-infectious trajectories such as persistence, chronicity, recurrence, or relapse in a defined viral cohort, and therefore was not aligned with the study objective focusing on post-viral urticaria trajectories. |
| Liutu M, Kalimo K, Uksila J, Kalimo H. Etiologic aspects of chronic urticaria. Int J Dermatol. 1998;37:515–519. | Observational study evaluating potential etiological factors, including infections, autoimmune conditions, and immunologic abnormalities, in patients with pre-existing chronic urticaria. Although infections were identified in a subset of patients, these were predominantly bacterial and were not analyzed as a primary exposure leading to urticaria onset. The study focused on established chronic disease rather than incident urticaria following infection and did not provide stratified analysis or extractable long-term outcomes specifically following viral infection. It did not assess post-infectious trajectories such as persistence, chronicity, recurrence, or relapse in a defined viral cohort, and therefore was not aligned with the study objective focusing on post-viral urticaria trajectories. |
| Loricera J, Calvo-Río V, Mata C, Ortiz-Sanjuán F, González-López MA, Alvarez L, González-Vela MC, Armesto S, Fernández-Llaca H, Rueda-Gotor J, González-Gay MA, Blanco R. Urticarial vasculitis in Northern Spain: clinical study of 21 cases. Medicine (Baltimore). 2014;93:53–60. | Retrospective study evaluating clinical features, treatment, and outcomes of patients with urticarial vasculitis, a distinct clinicopathological entity characterized by urticarial-like lesions with histologic evidence of leukocytoclastic vasculitis. Although infections, including upper respiratory tract infections, were identified as potential precipitating factors in a subset of cases, the study population did not represent urticaria as defined in the inclusion criteria and included mixed aetiologies without stratified analysis of viral triggers. It did not assess urticaria occurring after viral infection or report post-infectious trajectories such as persistence, chronicity, recurrence, or relapse in a defined viral cohort, and therefore was not aligned with the study objective focusing on post-viral urticaria trajectories. |
| Lu L, Cao L, Zhang J, Lin B. Cutaneous manifestations associated with COVID-19 infection at a university hospital in eastern China. Am J Clin Exp Immunol. 2024;13:117–132. | Retrospective cross-sectional study evaluating a broad spectrum of cutaneous manifestations associated with COVID-19 infection, including a subset of urticarial lesions. Although urticaria was reported among the dermatologic findings, it was not analyzed as a primary condition and was assessed alongside multiple heterogeneous skin diseases. The study focused on the acute presentation and timing of skin lesions following infection without providing stratified analysis or extractable long-term outcomes specifically for urticaria. It did not assess post-infectious trajectories such as persistence, chronicity, recurrence, or relapse in a defined viral urticaria cohort, and therefore was not aligned with the study objective focusing on post-viral urticaria trajectories. |
| Lugović-Mihić L, Bukvić I, Bulat V, Japundžić I. Factors contributing to chronic urticaria/angioedema and nummular eczema resolution – which findings are crucial? Acta Clin Croat. 2019;58:595–603. | Retrospective study evaluating the influence of associated diseases and infections on treatment outcomes in patients with pre-existing chronic urticaria and nummular eczema. Although infections were identified, these were predominantly non-viral (e.g., Helicobacter pylori and urogenital infections) and were not analyzed as primary exposures leading to urticaria onset. The study focused on disease resolution following treatment of comorbid conditions rather than urticaria occurring after viral infection and did not provide stratified analysis or extractable long-term outcomes specifically following viral infection. It did not assess post-infectious trajectories such as persistence, chronicity, recurrence, or relapse in a defined viral cohort, and therefore was not aligned with the study objective focusing on post-viral urticaria trajectories. |
| Lutz D, Ramsey A, Conn K, Mustafa SS. A 5-year retrospective review of outpatient graded drug challenges. J Allergy Clin Immunol Pract. 2019. | Retrospective study evaluating the safety and outcomes of graded drug challenges in patients with suspected drug allergies. Although urticaria was reported among historical allergic reactions, it was not investigated as a primary condition and was assessed only in the context of drug hypersensitivity. The study did not examine urticaria occurring after viral infection or provide stratified analysis or extractable long-term outcomes such as persistence, chronicity, recurrence, or relapse. It did not assess post-infectious trajectories in a defined viral urticaria cohort, and therefore was not aligned with the study objective focusing on post-viral urticaria trajectories. |
| Mareri A, Adler SP, Nigro G. Herpesvirus-associated acute urticaria: an age-matched case-control study. PLoS ONE. 2013;8:e85378. | Case-control study evaluating the association between herpesvirus infections and acute or recurrent acute urticaria in children. Although viral infections were identified as significant triggers, the study focused on etiological associations and predominantly involved acute urticaria presentations. Despite follow-up, outcomes were limited to the occurrence of acute or recurrent episodes without providing stratified analysis or extractable long-term outcomes specifically following viral infection. It did not assess post-infectious trajectories such as persistence, chronicity, recurrence, or relapse beyond the acute phase in a defined viral cohort, and therefore was not aligned with the study objective focusing on post-viral urticaria trajectories. |
| McMahon DE, Amerson E, Rosenbach M, et al. Cutaneous reactions reported after Moderna and Pfizer COVID-19 vaccination: A registry-based study of 414 cases. J Am Acad Dermatol. 2021;85:46–55. | Evaluated cutaneous reactions, including urticarial eruptions, following mRNA COVID-19 vaccination in a registry-based case series, rather than urticaria associated with viral infection. Outcomes were limited to short-term onset, duration, and recurrence between vaccine doses, without assessment of long-term outcomes such as persistence, chronicity, recurrence, or relapse of urticaria beyond the acute phase. |
| Netchiporouk E, Sasseville D, Moreau L, et al. Evaluating comorbidities, natural history, and predictors of early resolution in a cohort of children with chronic urticaria. JAMA Dermatol. 2017;153:1236–1242. | Prospective cohort study evaluating the natural history, comorbidities, and predictors of resolution in children with pre-existing chronic urticaria. Although long-term outcomes such as disease resolution were assessed, the study did not evaluate urticaria occurring after viral infection and did not define viral infection as the exposure of interest. Therefore, it was not aligned with the study objective focusing on post-viral urticaria trajectories. |
| Orlova E, Smirnova L, Nesvizhsky Y, et al. Acute urticaria in children: course of the disease, features of skin microbiome. Adv Dermatol Allergol. 2022;39:164–170. | Observational study evaluating the clinical characteristics and skin microbiome in children with acute urticaria. Although infections were described as potential triggers, the study focused on acute-phase disease and severity without longitudinal follow-up. It did not assess long-term outcomes such as persistence, chronicity, recurrence, or relapse following viral infection, and therefore was not aligned with the study objective focusing on post-viral urticaria trajectories. |
| Plumb J, Norlin C, Young PC. Exposures and outcomes of children with urticaria seen in a pediatric practice-based research network: a case-control study. Arch Pediatr Adolesc Med. 2001;155:1017–1021. | Case-control study evaluating clinical features, exposures, and short-term outcomes of acute urticaria in children in primary care settings. Although viral illness was identified as a commonly suspected trigger, the study focused exclusively on acute urticaria with follow-up limited to 30 days and did not assess long-term outcomes beyond the initial episode. It did not evaluate persistence, chronicity, recurrence, or relapse of urticaria following viral infection, and therefore was not aligned with the study objective focusing on post-viral urticaria trajectories. |
| Pourali SP, Kohn AH, Jones ME, et al. Chronic spontaneous urticaria: a 16-year analysis of pediatric patient demographics, treatment patterns, and comorbidities. Dermatol Online J. 2021;27(8). | Cross-sectional study evaluating demographics, comorbidities, and treatment patterns among pediatric patients with pre-existing chronic spontaneous urticaria using a national ambulatory database. Although infections were reported as comorbid conditions, the study did not assess urticaria occurring after viral infection or evaluate temporal relationships between infection and disease onset. It also did not report long-term outcomes such as persistence, recurrence, or relapse in a post-infectious context, and therefore was not aligned with the study objective focusing on post-viral urticaria trajectories. |
| Riddler SA, Moodie Z, Clark J, et al. High frequency of chronic urticaria following an investigational HIV-1 BG505 MD39.3 trimer mRNA vaccine in a phase 1, randomized, open-label clinical trial (HVTN 302). Ann Intern Med. 2025. | Evaluated chronic urticaria following administration of an investigational HIV-1 mRNA vaccine in a clinical trial, rather than urticaria associated with viral infection. Although long-term outcomes such as persistence and chronicity were reported, the exposure of interest was vaccine-related rather than infection-related, and therefore not aligned with the study objective focusing on post-viral urticaria trajectories. |
| Rossi CM, Lenti MV, Merli S, et al. Omega 5-gliadin allergy in patients with recurrent acute urticaria. Eur Ann Allergy Clin Immunol. 2025;57:45–48. | Retrospective study evaluating the prevalence and clinical features of omega-5 gliadin (wheat) allergy in patients with recurrent acute urticaria. Although recurrent episodes were reported, the study focused on food allergy–related urticaria rather than viral infection as the exposure of interest. Urticaria was characterized by intermittent acute episodes rather than persistent or chronic disease, and the study did not assess long-term outcomes such as persistence, chronicity, recurrence, or relapse in a post-infectious context. Therefore, it was not aligned with the study objective focusing on post-viral urticaria trajectories. |
| Sackesen C, Sekerel BE, Orhan F, et al. The etiology of different forms of urticaria in childhood. Pediatr Dermatol. 2004;21:102–108. | Prospective study evaluating the etiological factors and clinical characteristics of acute, recurrent, and chronic urticaria in children. Although infections were frequently identified as potential triggers, these were predominantly bacterial and were not analyzed as a primary exposure leading to urticaria onset. The study focused on identifying causes rather than evaluating post-infectious disease trajectories and did not provide stratified analysis or extractable long-term outcomes such as persistence, chronicity, recurrence, or relapse following viral infection. Therefore, it was not aligned with the study objective focusing on post-viral urticaria trajectories. |
| Sahiner UM, Civelek E, Tuncer A, Yavuz ST, Karabulut E, Sackesen C, Sekerel BE. Chronic urticaria: etiology and natural course in children. Int Arch Allergy Immunol. 2011;156:224–230. | Retrospective study evaluating the etiology and natural course of pre-existing chronic spontaneous urticaria in children. Although long-term outcomes such as remission and relapse were assessed, viral infections were not confirmed as a causative trigger and the study did not evaluate urticaria occurring after viral infection. Therefore, it was not aligned with the study objective focusing on post-viral urticaria trajectories. |
| Sánchez J, Álvarez L, Cardona R. Prospective analysis of clinical evolution in chronic urticaria: Persistence, remission, recurrence, and pruritus alone. World Allergy Organ J. 2022;15:100705. | Evaluated the natural history and clinical evolution of chronic spontaneous urticaria in a prospective cohort, rather than urticaria associated with viral infection. Although long-term outcomes such as persistence, remission, and recurrence were reported, the exposure of interest was not infection-related, and therefore not aligned with the study objective focusing on post-viral urticaria trajectories. |
| Santa C, Valente CL, Mesquita M, et al. Acute urticaria in children: from pediatric Emergency Department to allergology consultation at a Central Hospital. Eur Ann Allergy Clin Immunol. 2022;54:168–174. | Retrospective study evaluating the epidemiology, suspected triggers, and management of acute urticaria in children presenting to an emergency department. Although infections were identified as common triggers, the study focused exclusively on acute presentations and short-term allergy evaluation without longitudinal follow-up. It did not assess long-term outcomes such as persistence, chronicity, recurrence, or relapse following viral infection, and therefore was not aligned with the study objective focusing on post-viral urticaria trajectories. |
| Segal N, Levy Y, Katz J, Danon YL. Dental caries in children with chronic idiopathic urticaria in Israel. Pediatr Asthma Allergy Immunol. 2001;15:43–47. | Cross-sectional study evaluating the association between dental caries and pre-existing chronic idiopathic urticaria in children. The study focused on oral health as a potential contributing factor and did not assess urticaria occurring after viral infection or evaluate temporal relationships between infection and disease onset. It also did not report long-term outcomes such as persistence, chronicity, recurrence, or relapse in a post-infectious context, and therefore was not aligned with the study objective focusing on post-viral urticaria trajectories. |
| Shin M, Lee S. Prevalence and causes of childhood urticaria. Allergy Asthma Immunol Res. 2017;9:189–190. | Narrative editorial summarizing the epidemiology and common triggers of urticaria in children. Although infections were described as frequent triggers, the article did not provide primary data or evaluate long-term outcomes such as persistence, chronicity, recurrence, or relapse following viral infection. Therefore, it was not aligned with the study objective focusing on post-viral urticaria trajectories. |
| Silvares MRC, Coelho KIR, Dalben I, et al. Sociodemographic and clinical characteristics, causal factors and evolution of a group of patients with chronic urticaria-angioedema. Sao Paulo Med J. 2007;125:281–285. | Prospective descriptive study evaluating clinical characteristics, causal factors, and disease evolution in patients with pre-existing chronic urticaria-angioedema. Although infections were identified as potential contributing factors, these were not limited to viral causes and were not analyzed as a primary exposure leading to urticaria onset. The study focused on the general natural history and treatment response of established chronic urticaria rather than urticaria occurring after viral infection, and did not provide stratified or extractable long-term outcomes in a post-infectious context. Therefore, it was not aligned with the study objective focusing on post-viral urticaria trajectories. |
| Simons FER, et al. Prevention of acute urticaria in young children with atopic dermatitis. J Allergy Clin Immunol. 2001;107:703–706. | Evaluated the effect of cetirizine treatment on the prevention of acute urticaria episodes in children with atopic dermatitis in a randomized controlled trial, without assessment of viral infection as the exposure of interest or urticaria occurring after infection. The study did not report long-term outcomes such as persistence, chronicity, recurrence, or relapse following infection, and therefore was not aligned with the study objective focusing on post-viral urticaria trajectories. |
| Sørensen HT, Christensen B, Kjærulff E. A two-year follow-up of children with urticaria in general practice. Scand J Prim Health Care. 1987;5:24–26. | Prospective follow-up study evaluating the clinical course and recurrence of urticaria in children in a general practice setting. Although long-term outcomes such as recurrence and duration were reported, the study included an unselected population of urticaria cases and did not assess viral infection as a defined exposure. Infection was not systematically evaluated or analyzed as a causal factor, and no stratified or extractable outcomes were reported for urticaria following viral infection. Therefore, it was not aligned with the study objective focusing on post-viral urticaria trajectories. |
| Stinco M, Bartolini E, Veronese P, et al. Epidemiology and natural history of childhood-acquired chronic hepatitis C: a single-center long-term prospective study. J Pediatr Gastroenterol Nutr. 2022;75:e2–e7. | Prospective cohort study evaluating the epidemiology and long-term outcomes of children with chronic hepatitis C infection. Although chronic urticaria was reported as a rare extrahepatic manifestation in a small number of patients, urticaria was not the primary condition under investigation and no data were provided on its clinical course or long-term outcomes. The study did not evaluate urticaria occurring after viral infection in a defined cohort or assess outcomes such as persistence, chronicity, recurrence, or relapse, and therefore was not aligned with the study objective focusing on post-viral urticaria trajectories. |
| Techasatian L, Phungoen P, Chaiyarit J, Uppala R. Etiological and predictive factors of pediatric urticaria in an emergency context. BMC Pediatr. 2021;21:92. | Cross-sectional study evaluating the etiological factors and associated clinical characteristics of pediatric urticaria in an emergency department setting. Although infections were identified as the most common trigger, the study did not include longitudinal follow-up or assess long-term outcomes such as persistence, chronicity, recurrence, or relapse. Therefore, it was not aligned with the study objective focusing on post-viral urticaria trajectories. |
| Turk M, Baysal B, Ertas R, et al. Listening to urticaria patients through social media: analyzing the impact of the COVID-19 pandemic. Asthma Allergy Immunol. 2025;23:86–95. | Social media listening study analyzing patient-reported experiences, symptoms, and perceptions of urticaria on digital platforms. The study did not involve a clinically defined cohort or evaluate urticaria following viral infection, and did not report longitudinal outcomes such as persistence, chronicity, recurrence, or relapse. Therefore, it was not aligned with the study objective focusing on post-viral urticaria trajectories. |
| Yilmaz EA, Karaatmaca B, Cetinkaya PG, et al. The persistence of chronic spontaneous urticaria in childhood is associated with the urticaria activity score. Allergy Asthma Proc. 2017;38:136–142. | Cohort study evaluating the natural history and persistence of chronic spontaneous urticaria in children, without assessment of urticaria occurring after viral infection. Although longitudinal outcomes such as remission and persistence were reported, the study population consisted of pre-existing chronic urticaria cases rather than infection-associated onset, and therefore was not aligned with the study objective focusing on post-viral urticaria trajectories. |
| Zhang B, Song H, Shen Q, et al. Detection of streptococcus pyogenes antibodies in acute idiopathic urticaria. Int J Clin Exp Med. 2017;10:10736–10741. | Observational study evaluating streptococcal antibody levels in patients with acute idiopathic urticaria. Although an association with bacterial infection markers was explored, the study focused on acute-phase disease without longitudinal follow-up. It did not assess long-term outcomes such as persistence, chronicity, recurrence, or relapse, and therefore was not aligned with the study objective focusing on post-viral urticaria trajectories. |
