# Supplementary material for: Persistence, chronicity, and recurrence of infection-associated urticaria following viral infections in children and adults: a systematic review
Source: Front Allergy. 2026 May 26;7:1847423. doi: 10.3389/falgy.2026.1847423 (PMC13246635; doi:10.3389/falgy.2026.1847423)
Supplement: Supplementary file 3 [file Table3.docx]

Supplementary Table S3. Detailed Newcastle–Ottawa Scale (NOS) scoring.

| Study | Representativeness | Selection of controls | Exposure ascertainment | Comparability | Outcome assessment | Follow-up adequacy | Total |
| --- | --- | --- | --- | --- | --- | --- | --- |
| Aoki (1994) [[7](#_ENREF_7)] | Cohort of patients with AU presenting within 7 days of onset at a dermatology clinic (moderately representative clinical population) | Not applicable (no control group) | Exposure (infection-related symptoms) assessed through detailed clinical history without laboratory confirmation of specific pathogens | No control group; no adjustment for confounders | Outcomes assessed through scheduled follow-up visits and telephone follow-up (duration and persistence of urticaria recorded) | Adequate (follow-up up to 1 year with outcome ascertainment reported) | 5/9 |
| Cetinkaya (2019) [[8](#_ENREF_8)] | Cohort of preschool children with AU recruited from pediatric allergy and emergency departments (moderately representative clinical population) | Not applicable (no control group) | Clinical assessment supported by laboratory investigations (viral serology, microbiological testing) | No control group; limited adjustment for confounders (risk factor analysis performed but not designed for causal inference) | Outcomes assessed through follow-up visits and telephone follow-up (recurrence and chronicity recorded) | Adequate (follow-up up to 2 years with outcome ascertainment reported) | 6/9 |
| Kara (2024) [[6](#_ENREF_6)] | Cohort of patients with urticaria following COVID-19 infection identified from specialized urticaria referral centres (moderately representative clinical population) | Comparison between acute urticaria and chronic spontaneous urticaria groups within the same cohort; no external control group | COVID-19 infection confirmed primarily by PCR testing, with clinical diagnosis in a minority of cases | Limited adjustment for confounders; comparisons performed between CSU and AU groups without multivariable adjustment | Outcomes (duration, chronicity, timing of onset) assessed through retrospective review of medical records | Moderate; follow-up duration sufficient to assess chronicity (up to ~22 months), but small CSU sample and retrospective design | 6/9 |
| Mortureux (1998) [[9](#_ENREF_9)] | Selected cohort of hospitalized infants with AU (not fully representative of general population) | Not applicable (no control group) | Clinical diagnosis supported by laboratory investigations (viral cultures, serology) | No adjustment for confounders or control group | Outcome assessed via follow-up visits and parental questionnaire | Moderate (follow-up available for 40/57 patients; ~70%) | 5/9 |
| Talarico (2021) [[5](#_ENREF_5)] | Cohort of children presenting with acute urticaria to multiple emergency departments across Italy (moderately representative clinical population) | Not applicable (no control group) | Exposure (infection-related trigger) assessed primarily through clinical history and physical examination; laboratory investigations performed selectively without systematic confirmation of viral pathogens | Limited adjustment for confounders; analyses of potential predictors (e.g., age, etiology, severity) performed without multivariable adjustment for key confounding factors | Outcomes (persistence and recurrence of urticaria) assessed through scheduled follow-up visits and structured questionnaires at 1, 3, and 6 months | Adequate (prospective follow-up conducted at predefined intervals up to 6 months with outcome ascertainment reported) | 6/9 |

Abbreviation: AU, acute urticaria; COVID-19, coronavirus disease 2019; CSU, chronic spontaneous urticaria; PCR, polymerase chain reaction.
